# A Systematic Review and Meta-Analysis of Injection Site Reactions in Randomized-Controlled Trials of Biologic Injections

**DOI:** 10.1177/12034754231188444

**Published:** 2023-08-02

**Authors:** Patrick J. Kim, Rafael Paolo Lansang, Ron Vender

**Affiliations:** 1 12362 Faculty of Medicine, McMaster University, Hamilton, Canada

**Keywords:** immunology, dermatology

## Abstract

**Background:**

Biologic agents are emerging as an important treatment option for immune-mediated diseases. Injection site reactions following subcutaneous injection of biologic agents is not well described in the literature.

**Objective:**

To summarize injection site reaction data in phase 3 trials of all biologic agents.

**Methods:**

MEDLINE, Embase, and CENTRAL databases were systematically searched on February 8, 2022. Proportional meta-analysis was conducted to summarize injection site reaction prevalence for each biologic.

**Results:**

There were 158 articles included in the review. The most common types of injection site reactions were erythema (42.8%), unspecified reaction (23.3%), pain (12.4%), and pruritus (5.7%). No patients discontinued their treatment due to injection site reactions in 39 of the 48 studies that reported on discontinuation data. There were 16 biologics included in meta-analysis across 80 eligible studies. The biologics with the highest point prevalence of patients reporting injection site reactions were Canakinumab (15.5%; 294 patients), Dupilumab (11.4%; 1888 patients), Etanercept (11.4%; 4363 patients), and Ixekizumab (11.2%; 2205 patients). The biologics with the lowest point prevalence of injection site reactions were Risankizumab (0.8%; 707 patients), Brodalumab (1.3%; 1365 patients), Guselkumab (1.3%; 1852 patients), Secukinumab (1.9%; 1277 patients).

**Conclusions:**

The prevalence of injection site reaction in response to biologics ranges from 0.08 to 15.5%. Canakinumab, Dupilumab, Etanercept, and Ixekizumab had the highest prevalence of injection site reactions. Risankizumab, Brodalumab, Guselkumab, and Secukinumab had the lowest prevalence of injection site reactions. Recommendations are made regarding the improvement of adverse event reporting to better understand the epidemiology of injection site reactions.

## Introduction

Biologic agents are becoming increasingly used in the treatment of immune-mediated diseases such as psoriasis and rheumatoid arthritis.^1,2^ They are typically administered parenterally as intravenous infusions or subcutaneous injections.^3^ The most utilized types of biologics in are tumor necrosis alpha (TNF-α) inhibitors and interleukin (IL) inhibitors, particularly inhibitors of IL-23 and IL-17.^4^ Biologics are thought to have improved safety profiles in comparison to systemic immunosuppression due to their selective targeting of specific aspects of the immune system.^5^ Biologics are indicated in severe conditions requiring immunotherapy that are unresponsive to more affordable and less invasive treatments.^4,6^ Systemic adverse effects of biologics are rare and include increased risk of serious infection, increased incidence of malignancy, and hematologic disturbances.^7^ Injection site reactions are adverse events in reaction to a drug or its excipient injected subcutaneously and often include localized pain, pruritus, or erythema.^3^

However, evidence on injection site reactions specifically in biologic treatments is limited and reported incidence rates of injection site reactions are highly variable between different biologics. According to a narrative review conducted in 2019, incidence of injection site reactions vary greatly - between 0.5 to 40% depending on the biologic. The underlying mechanisms of injection site reactions were found to be either irritative reactions caused by the proinflammatory actions of the drugs or allergic reactions to the drug itself.^8^ Despite the mandatory collection and reporting of adverse events in randomized controlled trials, the methodological and reporting quality surrounding adverse events in contemporary clinical trials have been found to be inconsistent.^9^

The primary aim of this systematic review and meta-analysis is to pool epidemiological data of biologic injection site reactions to provide better estimates of injection site reaction prevalence. Secondary aims are to summarize the types of injection site reactions encountered and make recommendations for improving injection site reaction reporting in the literature. We also hope to provide future clinicians and patients with the tools to make informed decisions about biologic treatment.

## Methods

This study was conducted in accordance with the Preferred Reporting Items for Systematic Reviews and Meta-Analyses 2020 statement (Supplemental File 1).^10^ The review protocol was not registered.

### Search Strategy and Eligibility

A search was conducted on MEDLINE, Embase, and CENTRAL databases from database inception to February 8th, 2022. We chose to include biologics with and without primarily dermatologic indications to conduct a comprehensive review of all available data on injection site reactions. The search strategy combined [biologic names] with Boolean operator “OR”, which was combined with variations of [phase 3 clinical trial] with Boolean operator “AND” (Supplemental File 2).

The inclusion criteria included studies that: (1) were original phase 3 clinical trials; (2) trialed a biologic medication; (3) used injection as the form of medication administration; (4) had complete reporting of injection site reactions as adverse events; (5) were conducted on human participants. Secondary analyses of trials (e.g., post-hoc analysis, long-term extensions) or trials that administered biologics intravenously were excluded. References of included studies were checked manually to identify entries not included in the search. Grey literature (clinicaltrials.gov) was also searched to obtain original trial data of secondary trial analyses identified in the search.

### Study Selection and Data Extraction

Two reviewers (P.K. and P.L.) conducted title/abstract and full-text screening and data extraction in duplicate. Conflicts were reached either by discussion or by a third reviewer (R.V.). The following variables were extracted: study characteristics, biologic intervention details (type, dose, frequency, concomitant medications), injection site reactions, and comparator group data. Biologic dosing regimens were divided into 2 categories: U.S. Food and Drug Administration (FDA)-approved dosing or non-FDA-approved. Only biologics administered under FDA-approved dosing were included in meta-analysis.

The Medical Dictionary for Regulatory Activities (MedDRA), a dictionary of standardized medical terminology developed for coding of adverse effects, defines “injection site reactions” as a “high-level” grouping term with subclasses of “preferred terms” used to describe the type of reaction.^11,12^ Studies were categorized into: (1) studies that reported all injection site reactions under “injection site reactions”; (2) studies with reporting of all high-level injection site reactions and its preferred terms within; (3) studies that reported only the most common preferred terms. Category 3 studies were excluded from meta-analysis to avoid undercounting of injection site reactions due to the exclusion of less common preferred terms.

### Statistical Analysis

Descriptive statistics were used to summarize study characteristics, biologic indications, and types of injection site reactions. Median values were reported with interquartile ranges (IQR).

A proportional meta-analysis using a random-effects model was conducted to determine point estimates with 95% confidence intervals (CI) of the pooled prevalence of injection site reactions for each biologic as according to Barker et al.^13^ Meta-analysis was not conducted if there were <2 study arms investigating the biologic.

The I^2^ model was used to assess heterogeneity, where I^2^ >50% was significantly heterogenous. *P* value less than 0.05 was statistically significant.^14^ All meta-analyses were conducted using R Studio.^15^

## Results

**Figure 1:**
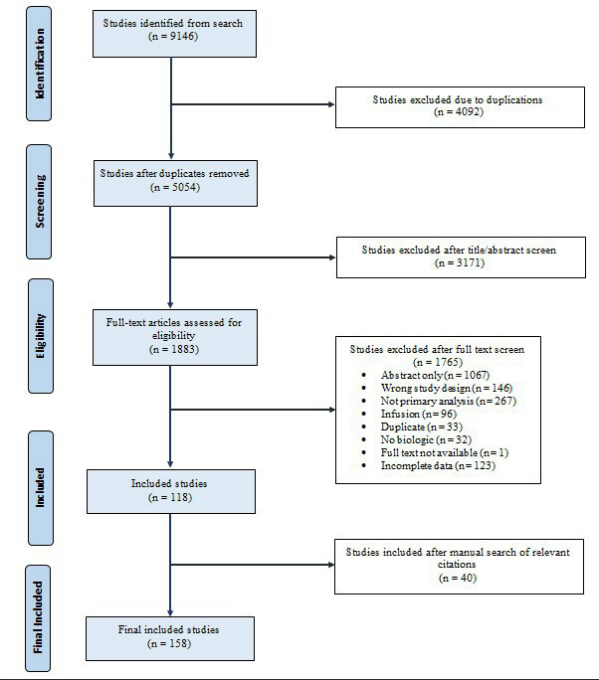
Selection process for study inclusion. A total of 158 articles were included for analysis at the end of the selection process for study inclusion.

There were 9146 studies identified in the search and 118 studies included for the review after screening (Figure 1). An additional 40 studies were included through a manual search for a total of 158 studies in this review. Publication dates of included studies were as follows: 1999 (0.6%, *n* = 1/158), 2000-2004 (2.5%, *n* = 4/158), 2005-2009 (10.8%, *n* = 17/158), 2010-2014 (22.8%, *n* = 36/158), 2015-2019 (39.8%, *n* = 63/158), 2020-2022 (23.4%, *n* = 37/158). Most studies involved investigators from multiple countries (77.2%, *n* = 122/158). There were 13.3% (*n* = 21/158) of studies conducted in exclusively in Japan, 3.8% (*n* = 6/159) conducted in the United States, 3.2% (*n* = 5/158) conducted in China, 1.3% (*n* = 2/158) conducted in Canada, and 0.6% (*n* = 1/158) each conducted in Germany and Russia (Supplemental File 3).

Biologics identified in our study were Abatacept, Adalimumab, Amgevita, Briakinumab, Brodalumab, Canakinumab, Certolizumab Pegol, Dupilumab, Etanercept, Golimumab, Guselkumab, Ixekizumab, Mepolizumab, Netakinumab, Omalizumab, Rilonacept, Risankizumab, Sarilumab, Secukinumab, Sirukumab, Tezepelumab, Tocilizumab, Ustekinumab, and Vedolizumab.

Details regarding injection site reaction preferred terms available for 1561 injection site reactions across in 71 studies (44.9%, *n* = 71/158). The most common injection site reaction was erythema (42.8%, *n* = 669/1561), followed by reaction (23.3%, *n* = 395/1561), pain (12.4%, *n* = 194/1561), and pruritus (5.7%, *n* = 89/1561). A full breakdown of injection site reactions preferred terms is available in Table 1. There were 48 (30.4%, *n* = 48/158) studies that reported on treatment discontinuation due to injection site reactions; 81.3% (*n* = 39/48) of studies had 0 patients discontinue treatment due to injection site reactions, 12.5% (*n* = 6/48) had 1 patient each, and 6.3% (*n* = 3/48) had 2 patients each.

**Table 1:** Injection Site Reactions by Preferred MedDRA Terms.

MedDRA preferred term	n/N (%)
Erythema	669/1561 (42.8%)
Reaction	395/1561 (23.3%)
Pain	194/1561 (12.4%)
Pruritus	89/1561 (5.7%)
Edema	35/1561 (2.2%)
Swelling	31/1561 (2.0%)
Ecchymosis	23/1561 (1.5%)
Irritation	21/1561 (1.3%)
Hematoma	19/1561 (1.2%)
Rash	19/1561 (1.2%)
Nodule	17/1561 (1.1%)
Coldness	14/1561 (0.9%)
Bruising	11/1561 (0.7%)
Induration	7/1561 (0.4%)
Hypersensitivity	6/1561 (0.4%)
Hemorrhage	5/1561 (0.3%)
Urticaria	2/1561 (0.1%)
Inflammation	1/1561 (0.1%)
Macule	1/1561 (0.1%)
Papule	1/1561 (0.1%)
Warmth	1/1561 (0.1%)

There were 80 studies (50.6%, *n* = 80/158) eligible for meta-analysis; 22 studies (13.9%, *n* = 22/158) were not eligible for meta-analysis due to the incomplete reporting of injection site reactions in their results and 56 studies (35.4%, *n* = 56/158) did not use FDA-approved dosing regimens.

### Adalimumab

There were 45 trials that investigated Adalimumab, of which there were 53 study arms. The most common preferred terms were erythema (28.7%, *n* = 45/157), reaction (20.4%, *n* = 32/157), pruritus (13.3%, *n* = 21/157), pain (12.1%, *n* = 19/157), and irritation (10.8%, *n* = 17/157). Four patients discontinued adalimumab due to injection site reactions. Management of injection site reactions was not reported in any of the trials.

**Figure 2:**
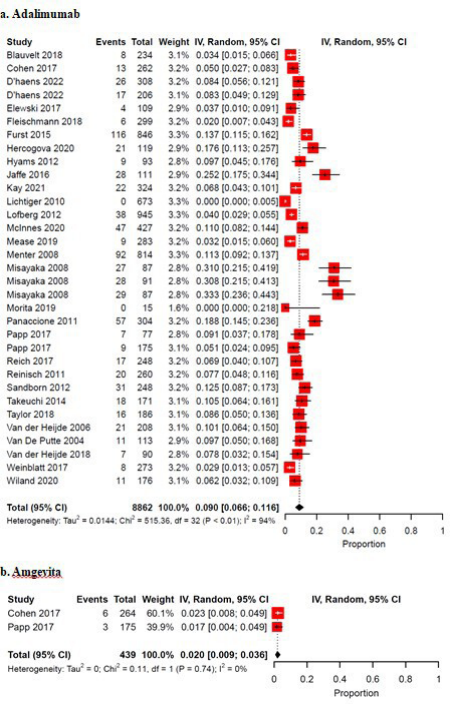
(a) Pooled prevalence of ISRs in randomized controlled trials investigating Adalimumab.(b) Pooled prevalence of ISRs in randomized controlled trials investigating Amgevita, an Adalimumab biosimilar. IV = inverse variance; CI = confidence interval.

For meta-analysis, there were 31 unique trials and 33 study arms eligible totaling 8862 patients. Indications for biologics were rheumatoid arthritis (RA) (36.4%, *n* = 12/33), plaque psoriasis (PsO) (21.2%, *n* = 7/33), Crohn’s disease (CD) (18.2%, *n* = 6/33), ankylosing spondylitis (AS) (6.1%, *n* = 2/33), psoriatic arthritis (PsA) (6.1%, *n* = 2/33), ulcerative colitis (UC) (6.1%, *n* = 2/33), hidradenitis suppurativa (3.0%, *n* = 1/33), and noninfectious uveitis (3.0%, *n* = 1/33). The median follow-up duration was 24 weeks (IQR: 16-26). The pooled prevalence of injection site reactions was 0.090 (95% CI: 0.066-0.116, I^2^: 94%) (Figure 2a).

There were 2 trials that studied Amgevita, an Adalimumab biosimilar with 439 patients across 2 study arms that were eligible for meta-analysis. There was no data available on injection site reaction type, discontinuation, or management. Indications for Amgevita were for RA and PsO. Follow-up duration was 16 weeks and 26 weeks. The pooled prevalence of injection site reactions was 0.020 (95% CI: 0.009-0.036, I^2^: 0%) (Figure 2b).

There were 9 other studies that investigated other Adalimumab biosimilars that were not eligible for meta-analysis.

### Certolizumab Pegol

**Figure 3:**
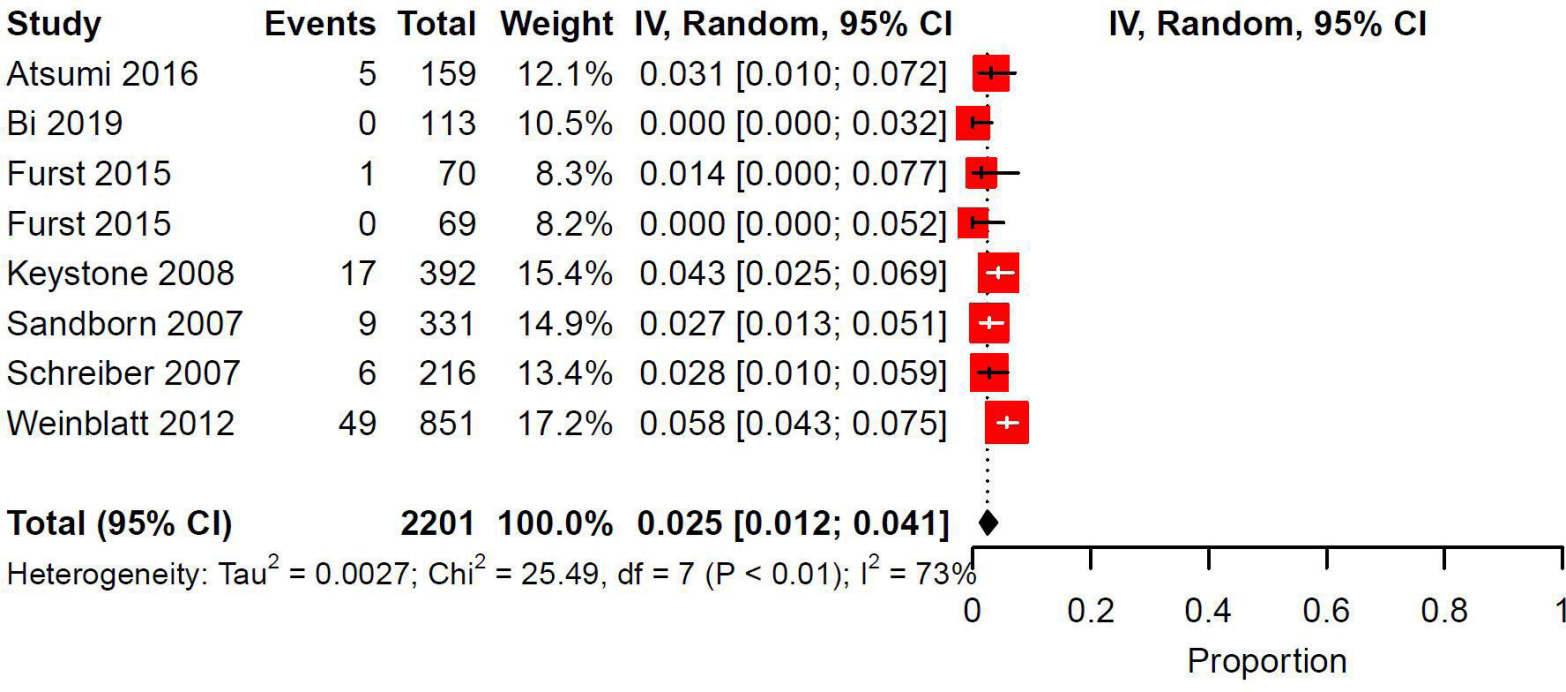
Pooled prevalence of ISRs in randomized controlled trials investigating Certolizumab Pegol. IV = inverse variance; CI = confidence interval.

Eleven trials and 15 study arms investigated Certolizumab pegol, for which injection site reaction preferred terms were available for 41 reactions. The reaction types were pain (51.2%, *n* = 21/41), reaction (36.6%, *n* = 15/41), erythema (7.3%, *n* = 3/41), rash (2.4%, *n* = 1/41), and hematoma (2.4%, *n* = 1/41). No studies reported on discontinuation or management. Meta-analysis was conducted on 2201 patients across 7 trials and 8 study arms. Indications for Certolizumab pegol were RA (75.0%, *n* = 6/8) and CD (25.0%, *n* = 2/8). Median follow-up duration was 24 weeks (IQR: 18-32.5). The pooled prevalence of injection site reactions was 0.025 (95% CI: 0.012-0.041, I^2^: 73%) (Figure 3).

### Etanercept

Etanercept was investigated in 20 trials and 28 study arms. Injection site reaction preferred terms were available for 179 reactions; the most common types were erythema (44.1%, *n* = 79/179), reaction (36.9%, *n* = 66/179), ecchymosis (12.8%, *n* = 23/179), and rash (3.4%, *n* = 6/179). There were 5 patients who discontinued etanercept due to injection site reactions. Management of injection site reactions were not reported.

**Figure 4:**
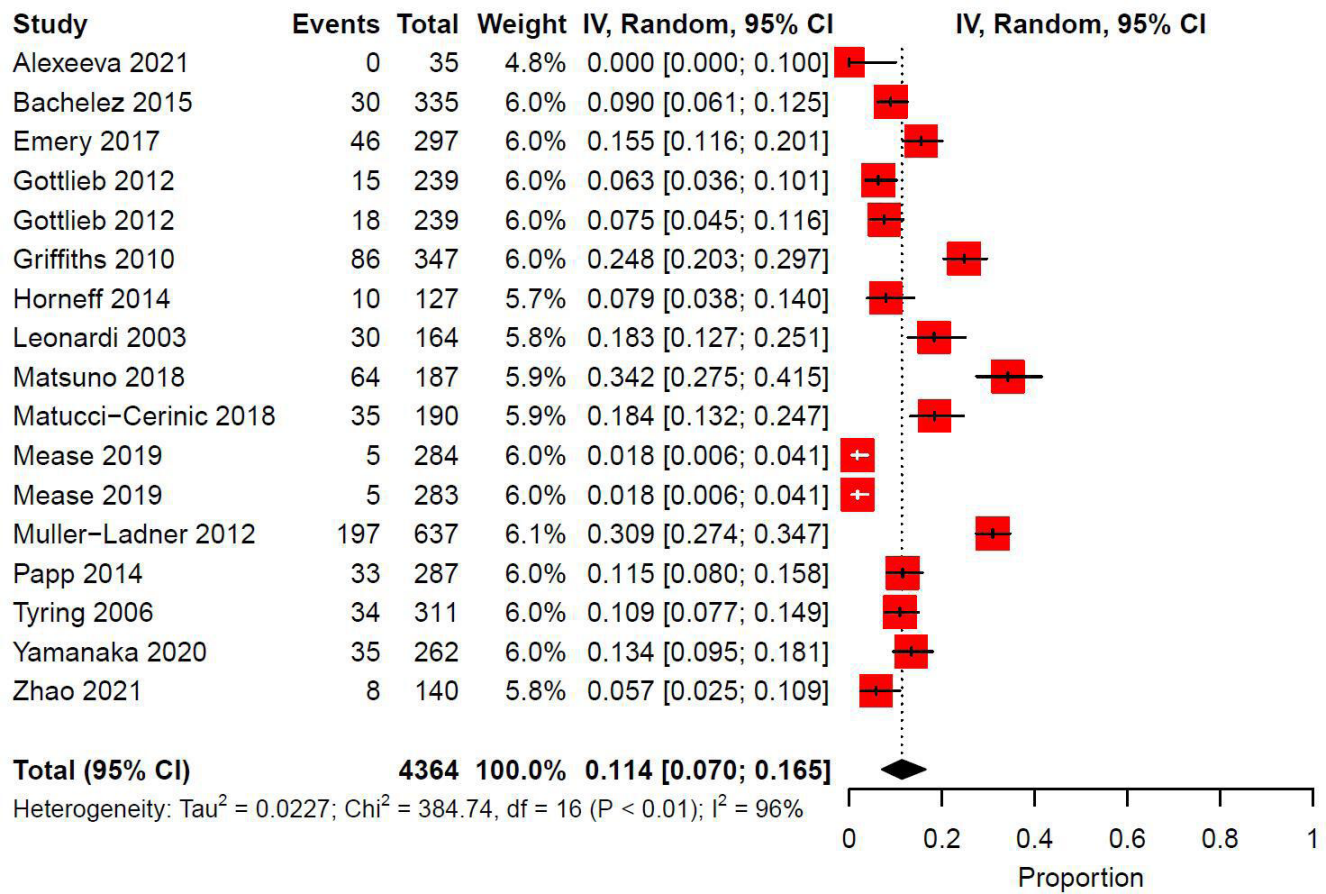
Pooled prevalence of ISRs in randomized controlled trials investigating Etanercept. IV = inverse variance; CI = confidence interval.

There were 15 trials and 17 study arms eligible for meta-analysis for a total of 4364 patients. Indications for biologics were PsO (41.2%, *n* = 7/17), RA (29.4%, *n* = 5/17) PsA (11.8%, *n* = 2/17), AS (5.9%, *n* = 1/17), JIA (5.9%, *n* = 1/17), and extended oligoarticular juvenile idiopathic arthritis (eoJIA)/enthesitis-related arthritis (ERA)/PsA (1/17). The median follow-up duration was 24 weeks (IQR: 12-24). Pooled prevalence of injection site reactions was 0.114 (95% CI: 0.070-0.165, I^2^: 96%) (Figure 4).

There were 5 Etanercept biosimilars across 5 trials not eligible for meta-analysis.

### Ixekizumab

**Figure 5:**
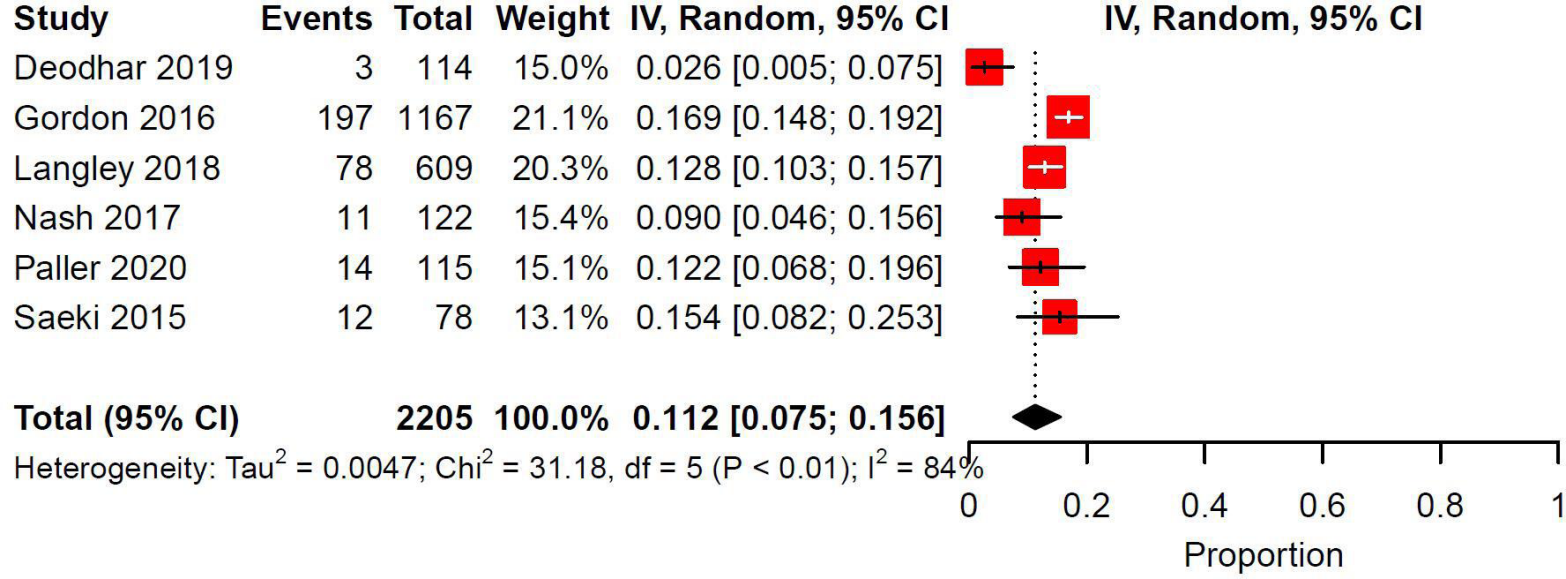
Pooled prevalence of ISRs in randomized controlled trials investigating Ixekizumab. IV = inverse variance; CI = confidence interval.

Eleven trials and 19 study arms investigated Ixekizumab. The most common injection site reaction types were reaction (58.7%, *n* = 229/390), erythema (28.2%, *n* = 110/390), pain (11.5%, *n* = 45/390), and hypersensitivity (1.5%, *n* = 6/390). There were 5 patients who discontinued Ixekizumab as a result of injection site reactions. Management of injection site reactions were not reported. Meta-analysis was conducted for 7 study arms across 7 trials for a total of 2205 patients. Indications for Ixekizumab were PsO (57.1%, *n* = 4/7), PsA (28.8%, *n* = 2/7), and axial spondylarthritis (14.3%, *n* = 1/7). Median follow-up duration was 24 weeks (IQR: 14-24). The pooled prevalence of injection site reactions was 0.112 (95% CI: 0.075-0.156, I^2^: 84%) (Figure 5).

### Ustekinumab

**Figure 6:**
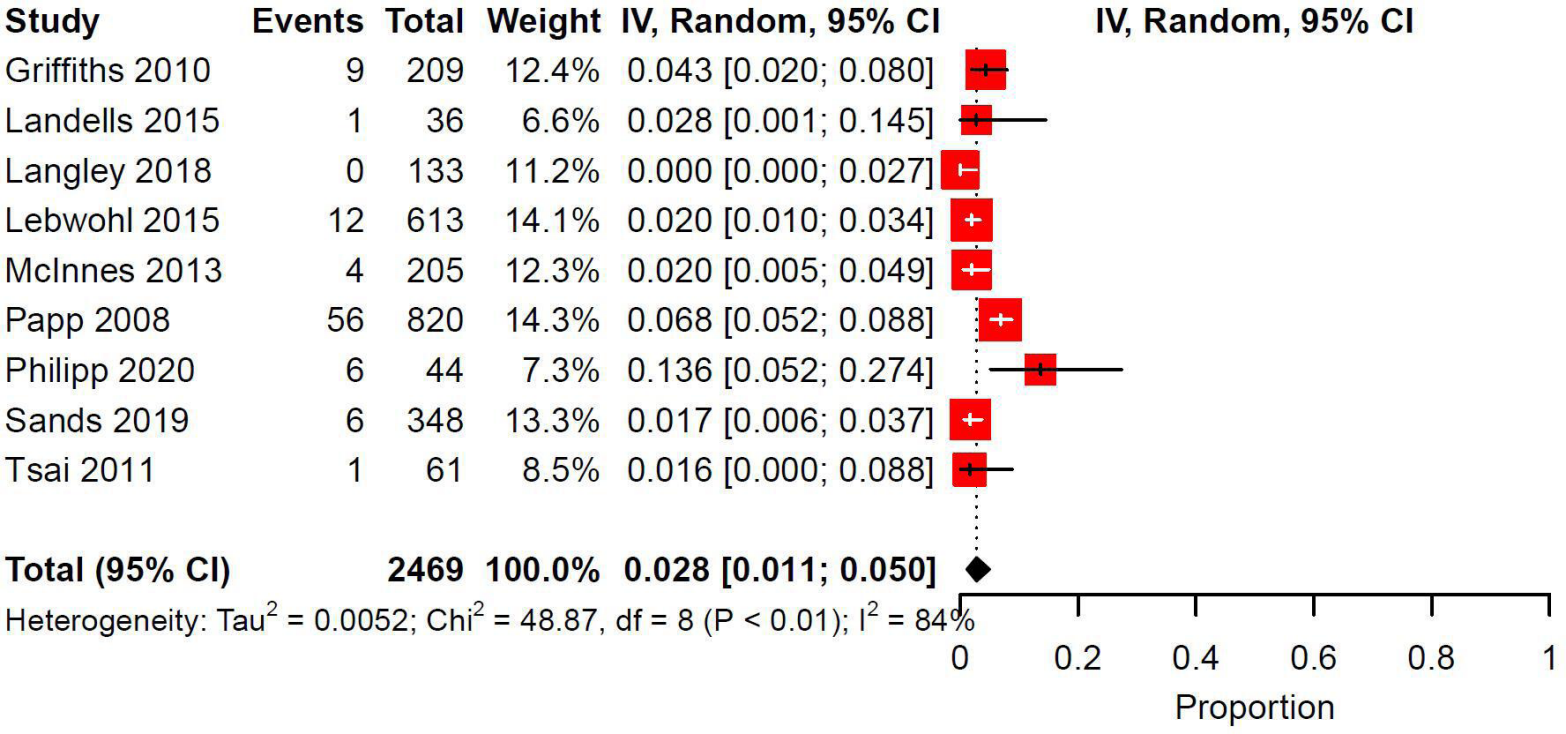
Pooled prevalence of ISRs in randomized controlled trials investigating Ustekinumab. IV = inverse variance; CI = confidence interval.

There were 9 trials with 12 study arms that investigated Ustekinumab. Injection site reaction descriptions were available for 12 reactions, all of which were erythema. No studies reported discontinuation due to injection site reactions. Management of injection site reactions were not reported. There were 9 trials with 9 study arms eligible for meta-analysis totaling 2469 patients. Indications for biologics were PsO (77.8%, *n* = 7/9), PsA (11.1%, *n* = 1/9), and UC (11.1%, *n* = 1/9). The median follow-up duration was 36 weeks (IQR: 12-52). The pooled prevalence of injection site reactions was 0.028 (95% CI: 0.011-0.050, I^2^: 84%) (Figure 6).

### Other Biologics

Other biologics with fewer than 2000 patients eligible for meta-analysis were Brodalumab (1365 patients), Canakinumab (294 patients), Dupilumab (1888 patients), Golimumab (875 patients), Guselkumab (1852 patients), Omalizumab (394 patients), Risankizumab (707 patients), Sarilumab (475 patients), Secukinumab (1277 patients), and Tocilizumab (1675 patients). Summary data for all biologics eligible for meta-analysis are available in Supplemental File 3. The forest plots are available in Supplemental File 4.

Biologics that were not eligible for meta-analysis were Abatacept, Briakinumab, Mepolizumab, Netakimab, Rilonacept, Sirukumab, Tezepelumab, and Vedolizumab.

## Discussion

This systematic review and meta-analysis summarized adverse event data of injection site reactions following biologic use across 158 phase 3 clinical trials. Adverse reactions to biologics have been categorized into 5 distinct types (type α, β, γ, δ and ɛ) according to Pichler et al., of which injection site reactions can be either type α or β.^16^ Type α reactions are caused by high levels of cytokines and typically cause systemic flu-like symptoms such as fever, fatigue, and myalgias.^17^ With subcutaneous injection of biologics locally irritative injection site reactions can occur due to the proinflammatory action of the injected substance causing elevated local cytokine concentrations.^8^

Type β reactions can be categorized into immediate (IgE-mediated) and delayed (IgG, complement, or *T* cell-mediated).^8^ Local subcutaneous injection of biologics can cause a rapid IgE-mediated allergic reaction, which presents as a local wheal and erythema, or urticaria/anaphylaxis in more severe reactions.^16^ Delayed IgG-mediated reactions present hours to days after administration and lead to inactivation of the biologic, causing patients to require greater doses.^16^ The most common types of injection site reactions in our review were erythema (42.8%), reaction (unspecified reaction; 23.3%), pain (12.4%), and pruritus (5.7%). One trial of Adalimumab in CD patients reported 5 accounts of injection site hemorrhage, but this was not elaborated upon in the results.^18^

Although injection site reactions are usually mild and rarely classified as severe adverse events, they may have an important impact on patient satisfaction with treatment and contribute to reasons for treatment discontinuation.^8^ A patient survey in 2015 of reasons for biologic discontinuation reported that the most common reason after lack of effectiveness was negative feelings about the injection experience.^19^ There are efforts being made to improve the experience for patients; notably, a new citrate-free formulation of Ixekizumab has shown a significant reduction in injection site pain in phase 1 trials.^20^ Thus, we may see improvements in injection site reaction prevalence in Ixekizumab or others that adopt similar solutions in the future.

Unfortunately, the reporting of reasons for discontinuation in biologic clinical trials frequently group all adverse events under one category, which makes this information difficult to extract. Most of our studies did not report on discontinuation due to injection site reactions. Among the 48 studies that did report this information, 81.3% of them had 0 patients discontinue their biologics as a result of injection site reactions, 12.5% had 1 patient each, and 6.3% had 2 patients each.

Meta-analysis was conducted for 50.6% of our studies for 16 different biologics (Table 2). There were 13.9% of studies excluded as they did not report a total number of injection site reactions and/or only reported the most common preferred term injection site reactions, which would have underestimated the pooled prevalence. The remaining 35.4% of studies did not investigate FDA-approved dosing regimens. The biologics with the highest prevalence of patients reporting injection site reactions were Canakinumab (15.5%; 294 patients), Dupilumab (11.4%; 1888 patients), Etanercept (11.4%; 4363 patients), and Ixekizumab (11.2%; 2205 patients). This is mostly in line with the 2019 review by Thomaidou et al.; Dupilumab was estimated to have a prevalence of 7 to 10%, Etanercept 2.97 to 37%, and Ixekizumab 13 to 17%.^8^ To our knowledge there is no summary data in the literature for Canakinumab.

**Table 2:** Summary of Prevalence of Injection Site Reactions for Biologics Eligible for Meta-Analysis.

Biologic name	Patients	Number of trials	Number of study arms	Prevalence (95% CI)
Adalimumab	8862	31	33	0.090 (0.066, 0.116)
Amgevita	439	2	2	0.020 (0.009, 0.036)
Brodalumab	1365	2	2	0.013 (0.007, 0.020)
Canakinumab	294	4	4	0.155 (0.036, 0.326)
Certolizumab Pegol	2201	7	8	0.025 (0.012, 0.041)
Dupilumab	1888	4	5	0.114 (0.068, 0.169)
Etanercept	4363	15	17	0.114 (0.070, 0.165)
Golimumab	875	5	6	0.035 (0.012, 0.069)
Guselkumab	1852	8	8	0.013 (0.003, 0.030)
Ixekizumab	2205	6	6	0.112 (0.075, 0.156)
Omalizumab	394	2	2	0.045 (0.000, 0.161)
Risankizumab	707	2	2	0.008 (0.002, 0.016)
Sarilumab	475	2	2	0.096 (0.071, 0.125)
Secukinumab	1277	4	4	0.019 (0.000, 0.059)
Tocilizumab	1675	5	5	0.069 (0.039, 0.106)
Ustekinumab	2469	9	9	0.028 (0.011, 0.050)

The biologics with the lowest prevalence of injection site reactions were Risankizumab (0.8%; 707 patients), Brodalumab (1.3%; 1365 patients), Guselkumab (1.3%; 1852 patients), Secukinumab (1.9%; 1277 patients). Again, this is mostly in line with the literature (Brodalumab 0.5 to 1.4%, Guselkumab 0.5 to 2.4%, Secukinumab < 1%, according to Thomaidou et al.).^8^ To our knowledge there is no summary data in the literature for Risankizumab.

It is worth noting that most studies that report data on injection site reactions use the term *incidence* as a misnomer; this is a commonly made mistake. Incidence defines the number of occurrences over a specified period of time (e.g., number of injection site reactions per year on the biologic), while *prevalence* is a proportion of patients affected by an event.^21^ Given the nature of adverse event reporting in clinical trials, which usually reports the number of patients affected rather than number of unique events, incidence data can rarely be calculated.^22^ Therefore in our meta-analysis we conducted a proportional meta-analysis to determine the pooled prevalence of injection site reactions. Unfortunately, this introduced a limitation to our review, as included studies had varying follow-up durations which affected our ability to pool accurate prevalence data. Studies with a longer duration of follow-up may have had greater prevalence compared to those with shorter follow-up due to the increased number of exposures to the biologic. However, there is evidence that suggests incidence of injection site reactions decrease over time, which may decrease the significance of this limitation.^22-24^

Another limitation is the heterogeneity of our meta-analysis findings. All estimates of prevalence were found to be significantly heterogenous (I^2^ >50%), which suggests that prevalence data may be inappropriately pooled. However, I^2^ statistics in proportional meta-analysis are usually high due to the low variance of proportional data, but no specific measure of heterogeneity has been developed for this type of analysis. Therefore Barker et al. recommends conservative interpretation of heterogeneity in that it does not necessarily indicate inconsistency in the data.^13^

This review also did not include studies that did not report on the presence nor absence of injection site reactions in any capacity, as it would be impossible to determine whether patients did not have any injection site reactions or there were not enough reactions to be reported in the study results. This resulted in certain biologics such as Bimekizumab being excluded from the review entirely. For example, the BE RADIANT trial by Reich *et al*. 2021 was excluded from the review as they reported only the top 5% most common adverse events and selected adverse events of interest, which did not include injection site reactions.^25^ This may have also biased the estimates of prevalence to be higher than the true prevalence.

Our data were also limited by the reporting of injection site reaction preferred terms in the included studies; less than half (44.9%) of studies reported preferred terms for injection site reactions. Additionally, among the preferred terms, “reaction” was the second most common, accounting for 23.3% of all reaction types. While “reaction” is a preferred term designated by MedDRA, describing injection site reactions as “reactions” gives no relevance to the extent, timing, or clinical relevance of the adverse event.^12^ Efforts should be made to more precisely articulate the nature of injection site reactions in clinical trials.

## Conclusion

The prevalence of injection site reaction in response to biologics varies widely from 0.08 to 15.5%. In our review, the biologics with the highest prevalence of injection site reactions were Canakinumab, Dupilumab, Etanercept, and Ixekizumab, although this may improve in the future with changes to biologic formulations. The biologics with the lowest prevalence of injection site reactions were Risankizumab, Brodalumab, Guselkumab, and Secukinumab.

We recommend future clinical trials investigating biologics implement more complete reporting of injection site reactions in terms of type, number of events, and changes in occurrence over time to facilitate a better understanding of its epidemiology and how it impacts patients who receive biologic medications.

## Supplemental Material

online supplementary file 1 - Supplemental material for A Systematic Review and Meta-Analysis of Injection Site Reactions in Randomized-Controlled Trials of Biologic Injections Click here for additional data file. Supplemental material, online supplementary file 1, for A Systematic Review and Meta-Analysis of Injection Site Reactions in Randomized-Controlled Trials of Biologic Injections by Patrick J. Kim, Rafael Paolo Lansang and Ron Vender in Journal of Cutaneous Medicine and Surgery

Online supplementary file 2 - Supplemental material for A Systematic Review and Meta-Analysis of Injection Site Reactions in Randomized-Controlled Trials of Biologic Injections Click here for additional data file. Supplemental material, Online supplementary file 2, for A Systematic Review and Meta-Analysis of Injection Site Reactions in Randomized-Controlled Trials of Biologic Injections by Patrick J. Kim, Rafael Paolo Lansang and Ron Vender in Journal of Cutaneous Medicine and Surgery

Online supplementary file 3 - Supplemental material for A Systematic Review and Meta-Analysis of Injection Site Reactions in Randomized-Controlled Trials of Biologic Injections Click here for additional data file. Supplemental material, Online supplementary file 3, for A Systematic Review and Meta-Analysis of Injection Site Reactions in Randomized-Controlled Trials of Biologic Injections by Patrick J. Kim, Rafael Paolo Lansang and Ron Vender in Journal of Cutaneous Medicine and Surgery

Online supplementary file 4 - Supplemental material for A Systematic Review and Meta-Analysis of Injection Site Reactions in Randomized-Controlled Trials of Biologic Injections Click here for additional data file. Supplemental material, Online supplementary file 4, for A Systematic Review and Meta-Analysis of Injection Site Reactions in Randomized-Controlled Trials of Biologic Injections by Patrick J. Kim, Rafael Paolo Lansang and Ron Vender in Journal of Cutaneous Medicine and Surgery
