# Supplementary material for: A Systematic Review and Meta-Analysis of Injection Site Reactions in Randomized-Controlled Trials of Biologic Injections
Source: J Cutan Med Surg. 2023 Aug 2;27(4):358–67. doi: 10.1177/12034754231188444 (PMC10486173; doi:10.1177/12034754231188444)
Supplement: Online supplementary file 2 - Supplemental material for A Systematic Review and Meta-Analysis of Injection Site Reactions in Randomized-Controlled Trials of Biologic Injections [file sj-docx-2-cms-10.1177_12034754231188444.docx]

**Database:**
OVID Medline Epub Ahead of Print, In-Process & Other Non-Indexed Citations, Ovid MEDLINE(R) Daily and Ovid MEDLINE(R) 1946 to Present

| **#** | **Query** | **Results from 9 Feb 2022** |
| --- | --- | --- |
| 1 | TNF [alpha.mp](http://alpha.mp/). [mp=title, abstract, original title, name of substance word, subject heading word, floating sub-heading word, keyword heading word, organism supplementary concept word, protocol supplementary concept word, rare disease supplementary concept word, unique identifier, synonyms] | 148,448 |
| 2 | [etanercept.mp](http://etanercept.mp/). [mp=title, abstract, original title, name of substance word, subject heading word, floating sub-heading word, keyword heading word, organism supplementary concept word, protocol supplementary concept word, rare disease supplementary concept word, unique identifier, synonyms] | 9,265 |
| 3 | [golimumab.mp](http://golimumab.mp/). [mp=title, abstract, original title, name of substance word, subject heading word, floating sub-heading word, keyword heading word, organism supplementary concept word, protocol supplementary concept word, rare disease supplementary concept word, unique identifier, synonyms] | 1,466 |
| 4 | [infliximab.mp](http://infliximab.mp/). [mp=title, abstract, original title, name of substance word, subject heading word, floating sub-heading word, keyword heading word, organism supplementary concept word, protocol supplementary concept word, rare disease supplementary concept word, unique identifier, synonyms] | 16,469 |
| 5 | [adalimumab.mp](http://adalimumab.mp/). [mp=title, abstract, original title, name of substance word, subject heading word, floating sub-heading word, keyword heading word, organism supplementary concept word, protocol supplementary concept word, rare disease supplementary concept word, unique identifier, synonyms] | 9,852 |
| 6 | IL 12 [23.mp](http://23.mp/). [mp=title, abstract, original title, name of substance word, subject heading word, floating sub-heading word, keyword heading word, organism supplementary concept word, protocol supplementary concept word, rare disease supplementary concept word, unique identifier, synonyms] | 467 |
| 7 | [ustekinumab.mp](http://ustekinumab.mp/). [mp=title, abstract, original title, name of substance word, subject heading word, floating sub-heading word, keyword heading word, organism supplementary concept word, protocol supplementary concept word, rare disease supplementary concept word, unique identifier, synonyms] | 2,595 |
| 8 | IL [17.mp](http://17.mp/). [mp=title, abstract, original title, name of substance word, subject heading word, floating sub-heading word, keyword heading word, organism supplementary concept word, protocol supplementary concept word, rare disease supplementary concept word, unique identifier, synonyms] | 19,338 |
| 9 | [brodalumab.mp](http://brodalumab.mp/). [mp=title, abstract, original title, name of substance word, subject heading word, floating sub-heading word, keyword heading word, organism supplementary concept word, protocol supplementary concept word, rare disease supplementary concept word, unique identifier, synonyms] | 438 |
| 10 | [ixekizumab.mp](http://ixekizumab.mp/). [mp=title, abstract, original title, name of substance word, subject heading word, floating sub-heading word, keyword heading word, organism supplementary concept word, protocol supplementary concept word, rare disease supplementary concept word, unique identifier, synonyms] | 786 |
| 11 | [secukinumab.mp](http://secukinumab.mp/). [mp=title, abstract, original title, name of substance word, subject heading word, floating sub-heading word, keyword heading word, organism supplementary concept word, protocol supplementary concept word, rare disease supplementary concept word, unique identifier, synonyms] | 1,576 |
| 12 | IL [23.mp](http://23.mp/). [mp=title, abstract, original title, name of substance word, subject heading word, floating sub-heading word, keyword heading word, organism supplementary concept word, protocol supplementary concept word, rare disease supplementary concept word, unique identifier, synonyms] | 6,363 |
| 13 | [guselkumab.mp](http://guselkumab.mp/). [mp=title, abstract, original title, name of substance word, subject heading word, floating sub-heading word, keyword heading word, organism supplementary concept word, protocol supplementary concept word, rare disease supplementary concept word, unique identifier, synonyms] | 395 |
| 14 | [risankizumab.mp](http://risankizumab.mp/). [mp=title, abstract, original title, name of substance word, subject heading word, floating sub-heading word, keyword heading word, organism supplementary concept word, protocol supplementary concept word, rare disease supplementary concept word, unique identifier, synonyms] | 243 |
| 15 | [tildrakizumab.mp](http://tildrakizumab.mp/). [mp=title, abstract, original title, name of substance word, subject heading word, floating sub-heading word, keyword heading word, organism supplementary concept word, protocol supplementary concept word, rare disease supplementary concept word, unique identifier, synonyms] | 189 |
| 16 | [certolizumab.mp](http://certolizumab.mp/). [mp=title, abstract, original title, name of substance word, subject heading word, floating sub-heading word, keyword heading word, organism supplementary concept word, protocol supplementary concept word, rare disease supplementary concept word, unique identifier, synonyms] | 1,461 |
| 17 | IL [1.mp](http://1.mp/). [mp=title, abstract, original title, name of substance word, subject heading word, floating sub-heading word, keyword heading word, organism supplementary concept word, protocol supplementary concept word, rare disease supplementary concept word, unique identifier, synonyms] | 40,286 |
| 18 | [anakinra.mp](http://anakinra.mp/). [mp=title, abstract, original title, name of substance word, subject heading word, floating sub-heading word, keyword heading word, organism supplementary concept word, protocol supplementary concept word, rare disease supplementary concept word, unique identifier, synonyms] | 2,268 |
| 19 | IL [4.mp](http://4.mp/). [mp=title, abstract, original title, name of substance word, subject heading word, floating sub-heading word, keyword heading word, organism supplementary concept word, protocol supplementary concept word, rare disease supplementary concept word, unique identifier, synonyms] | 47,513 |
| 20 | [dupilumab.mp](http://dupilumab.mp/). [mp=title, abstract, original title, name of substance word, subject heading word, floating sub-heading word, keyword heading word, organism supplementary concept word, protocol supplementary concept word, rare disease supplementary concept word, unique identifier, synonyms] | 1,491 |
| 21 | [omalizumab.mp](http://omalizumab.mp/). [mp=title, abstract, original title, name of substance word, subject heading word, floating sub-heading word, keyword heading word, organism supplementary concept word, protocol supplementary concept word, rare disease supplementary concept word, unique identifier, synonyms] | 3,152 |
| 22 | [mirikizumab.mp](http://mirikizumab.mp/). [mp=title, abstract, original title, name of substance word, subject heading word, floating sub-heading word, keyword heading word, organism supplementary concept word, protocol supplementary concept word, rare disease supplementary concept word, unique identifier, synonyms] | 26 |
| 23 | [vedolizumab.mp](http://vedolizumab.mp/). [mp=title, abstract, original title, name of substance word, subject heading word, floating sub-heading word, keyword heading word, organism supplementary concept word, protocol supplementary concept word, rare disease supplementary concept word, unique identifier, synonyms] | 1,410 |
| 24 | [natalizumab.mp](http://natalizumab.mp/). [mp=title, abstract, original title, name of substance word, subject heading word, floating sub-heading word, keyword heading word, organism supplementary concept word, protocol supplementary concept word, rare disease supplementary concept word, unique identifier, synonyms] | 2,918 |
| 25 | [canakinumab.mp](http://canakinumab.mp/). [mp=title, abstract, original title, name of substance word, subject heading word, floating sub-heading word, keyword heading word, organism supplementary concept word, protocol supplementary concept word, rare disease supplementary concept word, unique identifier, synonyms] | 927 |
| 26 | [tocilizumab.mp](http://tocilizumab.mp/). [mp=title, abstract, original title, name of substance word, subject heading word, floating sub-heading word, keyword heading word, organism supplementary concept word, protocol supplementary concept word, rare disease supplementary concept word, unique identifier, synonyms] | 5,423 |
| 27 | 1 or 2 or 3 or 4 or 5 or 6 or 7 or 8 or 9 or 10 or 11 or 12 or 13 or 14 or 15 or 16 or 17 or 18 or 19 or 20 or 21 or 22 or 23 or 24 or 25 or 26 | 265,911 |
| 28 | clinical trial, phase iii/ | 20,528 |
| 29 | 27 and 28 | 902 |

**Database:**
Embase <1974 to 2022 February 08>

| **#** | **Query** | **Results from 9 Feb 2022** |
| --- | --- | --- |
| 1 | TNF [alpha.mp](http://alpha.mp/). [mp=title, abstract, heading word, drug trade name, original title, device manufacturer, drug manufacturer, device trade name, keyword heading word, floating subheading word, candidate term word] | 189,067 |
| 2 | [etanercept.mp](http://etanercept.mp/). [mp=title, abstract, heading word, drug trade name, original title, device manufacturer, drug manufacturer, device trade name, keyword heading word, floating subheading word, candidate term word] | 35,471 |
| 3 | [golimumab.mp](http://golimumab.mp/). [mp=title, abstract, heading word, drug trade name, original title, device manufacturer, drug manufacturer, device trade name, keyword heading word, floating subheading word, candidate term word] | 8,643 |
| 4 | [infliximab.mp](http://infliximab.mp/). [mp=title, abstract, heading word, drug trade name, original title, device manufacturer, drug manufacturer, device trade name, keyword heading word, floating subheading word, candidate term word] | 57,771 |
| 5 | [adalimumab.mp](http://adalimumab.mp/). [mp=title, abstract, heading word, drug trade name, original title, device manufacturer, drug manufacturer, device trade name, keyword heading word, floating subheading word, candidate term word] | 40,083 |
| 6 | IL 12 [23.mp](http://23.mp/). [mp=title, abstract, heading word, drug trade name, original title, device manufacturer, drug manufacturer, device trade name, keyword heading word, floating subheading word, candidate term word] | 883 |
| 7 | [ustekinumab.mp](http://ustekinumab.mp/). [mp=title, abstract, heading word, drug trade name, original title, device manufacturer, drug manufacturer, device trade name, keyword heading word, floating subheading word, candidate term word] | 9,763 |
| 8 | IL [17.mp](http://17.mp/). [mp=title, abstract, heading word, drug trade name, original title, device manufacturer, drug manufacturer, device trade name, keyword heading word, floating subheading word, candidate term word] | 32,857 |
| 9 | [brodalumab.mp](http://brodalumab.mp/). [mp=title, abstract, heading word, drug trade name, original title, device manufacturer, drug manufacturer, device trade name, keyword heading word, floating subheading word, candidate term word] | 1,509 |
| 10 | [ixekizumab.mp](http://ixekizumab.mp/). [mp=title, abstract, heading word, drug trade name, original title, device manufacturer, drug manufacturer, device trade name, keyword heading word, floating subheading word, candidate term word] | 2,655 |
| 11 | [secukinumab.mp](http://secukinumab.mp/). [mp=title, abstract, heading word, drug trade name, original title, device manufacturer, drug manufacturer, device trade name, keyword heading word, floating subheading word, candidate term word] | 5,437 |
| 12 | IL [23.mp](http://23.mp/). [mp=title, abstract, heading word, drug trade name, original title, device manufacturer, drug manufacturer, device trade name, keyword heading word, floating subheading word, candidate term word] | 10,662 |
| 13 | [guselkumab.mp](http://guselkumab.mp/). [mp=title, abstract, heading word, drug trade name, original title, device manufacturer, drug manufacturer, device trade name, keyword heading word, floating subheading word, candidate term word] | 1,392 |
| 14 | [risankizumab.mp](http://risankizumab.mp/). [mp=title, abstract, heading word, drug trade name, original title, device manufacturer, drug manufacturer, device trade name, keyword heading word, floating subheading word, candidate term word] | 801 |
| 15 | [tildrakizumab.mp](http://tildrakizumab.mp/). [mp=title, abstract, heading word, drug trade name, original title, device manufacturer, drug manufacturer, device trade name, keyword heading word, floating subheading word, candidate term word] | 738 |
| 16 | [certolizumab.mp](http://certolizumab.mp/). [mp=title, abstract, heading word, drug trade name, original title, device manufacturer, drug manufacturer, device trade name, keyword heading word, floating subheading word, candidate term word] | 8,593 |
| 17 | IL [1.mp](http://1.mp/). [mp=title, abstract, heading word, drug trade name, original title, device manufacturer, drug manufacturer, device trade name, keyword heading word, floating subheading word, candidate term word] | 46,109 |
| 18 | [anakinra.mp](http://anakinra.mp/). [mp=title, abstract, heading word, drug trade name, original title, device manufacturer, drug manufacturer, device trade name, keyword heading word, floating subheading word, candidate term word] | 7,480 |
| 19 | IL [4.mp](http://4.mp/). [mp=title, abstract, heading word, drug trade name, original title, device manufacturer, drug manufacturer, device trade name, keyword heading word, floating subheading word, candidate term word] | 64,029 |
| 20 | [dupilumab.mp](http://dupilumab.mp/). [mp=title, abstract, heading word, drug trade name, original title, device manufacturer, drug manufacturer, device trade name, keyword heading word, floating subheading word, candidate term word] | 3,634 |
| 21 | [omalizumab.mp](http://omalizumab.mp/). [mp=title, abstract, heading word, drug trade name, original title, device manufacturer, drug manufacturer, device trade name, keyword heading word, floating subheading word, candidate term word] | 9,991 |
| 22 | [mirikizumab.mp](http://mirikizumab.mp/). [mp=title, abstract, heading word, drug trade name, original title, device manufacturer, drug manufacturer, device trade name, keyword heading word, floating subheading word, candidate term word] | 183 |
| 23 | [vedolizumab.mp](http://vedolizumab.mp/). [mp=title, abstract, heading word, drug trade name, original title, device manufacturer, drug manufacturer, device trade name, keyword heading word, floating subheading word, candidate term word] | 5,650 |
| 24 | [natalizumab.mp](http://natalizumab.mp/). [mp=title, abstract, heading word, drug trade name, original title, device manufacturer, drug manufacturer, device trade name, keyword heading word, floating subheading word, candidate term word] | 12,291 |
| 25 | [canakinumab.mp](http://canakinumab.mp/). [mp=title, abstract, heading word, drug trade name, original title, device manufacturer, drug manufacturer, device trade name, keyword heading word, floating subheading word, candidate term word] | 4,048 |
| 26 | [tocilizumab.mp](http://tocilizumab.mp/). [mp=title, abstract, heading word, drug trade name, original title, device manufacturer, drug manufacturer, device trade name, keyword heading word, floating subheading word, candidate term word] | 20,498 |
| 27 | 1 or 2 or 3 or 4 or 5 or 6 or 7 or 8 or 9 or 10 or 11 or 12 or 13 or 14 or 15 or 16 or 17 or 18 or 19 or 20 or 21 or 22 or 23 or 24 or 25 or 26 | 420,941 |
| 28 | phase 3 clinical trial/ | 60,413 |
| 29 | 27 and 28 | 3885 |

Search Name: CENTRAL database search

Date Run: 10/02/2022 05:34:31

Comment:

ID Search Hits

#1 etanercept 2437

#2 golimumab 800

#3 infliximab 2631

#4 adalimumab 3637

#5 ustekinumab 1025

#6 brodalumab 176

#7 ixekizumab 574

#8 secukinumab 1005

#9 guselkumab 374

#10 risankizumab 163

#11 tildrakizumab 190

#12 certolizumab 793

#13 anakinra 448

#14 dupilumab 636

#15 omalizumab 1002

#16 mirikizumab 105

#17 vedolizumab 494

#18 natalizumab 471

#19 canakinumab 355

#20 tocilizumab 1497

#21 #1 or #2 or #3 or #4 or #5 or #6 or #7 or #8 or #9 or #10 or #11 or #12 or #13 or #14 or #15 or #16 or #17 or #18 or #19 or #20 14838

#22 "phase 3" 49886

#23 "phase III" 51658

#24 #22 or #23 82917

#25 #21 and #24 in Trials 4359
