# Supplementary material for: A Systematic Review and Meta-Analysis of Injection Site Reactions in Randomized-Controlled Trials of Biologic Injections
Source: J Cutan Med Surg. 2023 Aug 2;27(4):358–67. doi: 10.1177/12034754231188444 (PMC10486173; doi:10.1177/12034754231188444)
Supplement: Online supplementary file 3 - Supplemental material for A Systematic Review and Meta-Analysis of Injection Site Reactions in Randomized-Controlled Trials of Biologic Injections [file sj-docx-3-cms-10.1177_12034754231188444.docx]

| **Study ID** | **Trial name/registration** | **Country** | **ISR reporting category** | **FDA-approved regimen** |
| --- | --- | --- | --- | --- |
| Alexeeva 2021^1^ | 2015–003384-11 | Russia | 1 | Y |
| Atsumi 2016^2^ | C-OPERA | Japan | 1 | Y |
| Bacharier 2021^3^ | LIBERTY ASTHMA VOYAGE | US | 3 | N |
| Bachelez 2015^4^ | OPT-COMPARE | multiple countries | 1 | Y |
| Bachert 2019^5^ | SINUS-24/SINUS-52 | US | 1 | Y |
| Bao 2014^6^ | NCT01248793 | China | 1 | Y |
| Bi 2019^7^ | RAPID-C | China | 1 | Y |
| Blauvelt 2018^8^ | NCT02016105 | multiple countries | 1 | Y |
| Bosch 2014^9^ | NCT01295814 | US | 1 | N |
| Brunner 2016^10^ | GO-KIDS | multiple countries | 1 | N |
| Burmester 2013^11^ | SUMMACTA | multiple countries | 1 | Y |
| Burmester 2017^12^ | MONARCH | multiple countries | 3 | Y |
| Callis Duffin 2017^13^ | UNCOVER-A | multiple countries | 3 | N |
| Castro 2018^14^ | LIBERTY ASTHMA QUEST | multiple countries | 2 | Y |
| Chen 2016^15^ | none | China | 1 | N |
| Chervinsky 2003^16^ | none | US | 1 | N |
| Chupp 2017^17^ | MUSCA | multiple countries | 1 | Y |
| Coates 2021^18^ | COSMOS | multiple countries | 1 | Y |
| Coates 2021^19^ | SPIRIT-P3 | multiple countries | 2 | N |
| Cohen 2017^20^ | NCT01970475 | multiple countries | 2 | Y |
| Cohen 2019^21^ | NCT02986139 | multiple countries | 3 | Y |
| Colombel 2007^22^ | CHARM | multiple countries | 3 | N |
| Colombel 2017^23^ | CALM | multiple countries | 3 | Y |
| D'agostino 2021^24^ | ULTIMATE | multiple countries | 1 | N |
| Debenedetti 2018^25^ | CLUSTER | multiple countries | 1 | Y |
| Debruin-weller 2018^26^ | LIBERTY AD CAFÉ | multiple countries | 3 | Y |
| Deodhar 2019^27^ | COAST-W | multiple countries | 1 | Y |
| Deodhar 2020^28^ | DISCOVER-1 | multiple countries | 1 | Y |
| D'haens 2022^29^ | SERENE CD | multiple countries | 1 | Y |
| Edwards 2019^30^ | AURIEL | multiple countries | 3 | Y |
| Elewski 2017^31^ | NCT02016482 | multiple countries | 1 | Y |
| Emery 2009^32^ | GO-BEFORE | multiple countries | 1 | Y |
| Emery 2017^33^ | NCT0189530 | multiple countries | 1 | Y |
| Emery 2019^34^ | ASCERTAIN | multiple countries | 1 | Y |
| Feldman 2021^35^ | NCT03849404 | multiple countries | 3 | Y |
| Ferris 2020^36^ | ORION | multiple countries | 3 | Y |
| Fleischmann 2018^37^ | REFLECTIONS B538-02 | multiple countries | 1 | Y |
| Furst 2015 | DE-020^38^ | multiple countries | 1 | Y |
| Furst 2015^39^ | DOSEFLEX | multiple countries | 1 | Y |
| Genovese 2015^40^ | RA-MOBILITY | multiple countries | 1 | Y |
| Gevaert 2020^41^ | POLYP-1-2 | multiple countries | 1 | N |
| Gordon 2016^42^ | UNCOVER-1-3 | multiple countries | 1 | Y |
| Gottlieb 2012^43^ | NCT01001208 | multiple countries | 1 | Y |
| Griffiths 2010^44^ | ACCEPT | multiple countries | 1 | Y |
| Hanania 2011^45^ | EXTRA | multiple countries | 3 | Y |
| Hercogova 2020^46^ | AURIEL-PsO | multiple countries | 1 | N |
| Hibi 2017^47^ | PURSUIT | Japan | 1 | N |
| Horneff 2014^48^ | CLIPPER | multiple countries | 1 | Y |
| Hyams 2012^49^ | IMAGINE-1 | multiple countries | 1 | Y |
| Imagawa 2013^50^ | none | Japan | 1 | Y |
| Inman 2008^51^ | GO-RAISE | multiple countries | 1 | Y |
| Jaffe 2016^52^ | VISUAL | multiple countries | 1 | Y |
| Kavanaugh 2009^53^ | NCT00265096 | multiple countries | 1 | Y |
| Kay 2021^54^ | NCT03789292 | multiple countries | 1 | Y |
| Keystone 2008^55^ | RAPID-1 | multiple countries | 1 | Y |
| Keystone 2009^56^ | GO-FORWARD | multiple countries | 1 | Y |
| Khanna 2020^57^ | focuSSed | multiple countries | 3 | N |
| Kingsbury 2014^58^ | NCT00775437 | multiple countries | 1 | N |
| Kivitz 2014^59^ | BREVACTA | multiple countries | 1 | Y |
| Kobayashi 2012^60^ | none | Japan | 3 | Y |
| Kristensen 2022^61^ | KEEPsAKE | multiple countries | 1 | Y |
| Kuehr 2002^62^ | none | multiple countries | 1 | N |
| Kuemmerle-Deschner 2011^63^ | NCT00685373 | multiple countries | 1 | Y |
| Landells 2015^64^ | CADMUS | multiple countries | 1 | Y |
| Landewe 2014^65^ | RAPID-axSpA | multiple countries | 3 | N |
| Langley 2018^66^ | IXORA-P | multiple countries | 1 | Y |
| Langley 2018^67^ | NAVIGATE | multiple countries | 1 | Y |
| Lebwohl 2015^68^ | AMAGINE-2-3 | multiple countries | 2 | Y |
| Leonardi 2003^69^ | none | multiple countries | 1 | Y |
| Li 2016^70^ | NCT01248780 | China | 1 | Y |
| Lichtiger 2010^71^ | CHOICE | US | 1 | Y |
| Lofberg 2012^72^ | CARE | multiple countries | 1 | Y |
| Magnolo 2022^73^ | NCT03668613 | multiple countries | 3 | Y |
| Matsuno 2018^74^ | NCT02357069 | multiple countries | 1 | Y |
| Matucci-Cerinic 2018^75^ | EQUIRA | multiple countries | 2 | Y |
| McInnes 2013^76^ | PSUMMIT-1 | multiple countries | 1 | Y |
| McInnes 2020^77^ | EXCEED | multiple countries | 2 | Y |
| Mease 2014^78^ | RAPID-PsA | multiple countries | 3 | Y |
| Mease 2017^79^ | SPIRIT-P1 | multiple countries | 3 | Y |
| Mease 2019^80^ | SEAM-PsA | multiple countries | 1 | Y |
| Mease 2019^81^ | SPIRIT-H2H | multiple countries | 1 | Y |
| Mease 2020^82^ | DISCOVER-2 | multiple countries | 1 | Y |
| Menter 2008^83^ | REVEAL | multiple countries | 1 | Y |
| Menter 2021^84^ | VOLTAIRE-PSO | multiple countries | 2 | Y |
| Menzies-Gow 2021^85^ | NAVIGATOR | multiple countries | 1 | Y |
| Miyasaka 2008^86^ | CHANGE | Japan | 1 | Y |
| Mitha 2013^87^ | PRESURGE-2 | multiple countries | 2 | N |
| Moreland 1999^88^ | none | multiple countries | 1 | N |
| Morita 2018^89^ | NCT02533375 | Japan | 1 | N |
| Morita 2019^90^ | NCT02904902 | Japan | 1 | Y |
| Motoya 2018^91^ | NCT01958827 | Japan | 1 | N |
| Muller-Ladner 2012^92^ | NCT00459706 | multiple countries | 1 | Y |
| Mysler 2020^93^ | TOZURA | multiple countries | 1 | Y |
| Nash 2017^94^ | SPIRIT-P2 | multiple countries | 1 | Y |
| Nash 2018^95^ | FUTURE-3 | multiple countries | 1 | Y |
| Nishimura 2021^96^ | NCT02396212 | Japan | 1 | Y |
| Ogata 2014^97^ | MUSASHI | Japan | 1 | Y |
| Ohtsuki 2018^98^ | NCT02325219 | Japan | 1 | Y |
| Ostor 2021^99^ | KEEPsAKE 2 | multiple countries | 1 | Y |
| Paller 2008^100^ | NCT00078819 | multiple countries | 1 | N |
| Paller 2020^101^ | IXORA-PEDS | multiple countries | 1 | Y |
| Paller 2020^102^ | LIBERTY AD PEDS | multiple countries | 2 | N |
| Panaccione 2011^103^ | ACCESS | Canada | 1 | Y |
| Papp 2005^104^ | none | multiple countries | 3 | Y |
| Papp 2008^105^ | PHOENIX 2 | multiple countries | 1 | Y |
| Papp 2014^106^ | REFINE | Canada | 1 | Y |
| Papp 2016^107^ | AMAGINE 1 | multiple countries | 1 | Y |
| Papp 2017^108^ | NCT01251614 | multiple countries | 1 | Y |
| Papp 2017^109^ | NCT01970488 | multiple countries | 1 | Y |
| Paul 2015^110^ | JUNCTURE | multiple countries | 2 | Y |
| Philipp 2020^111^ | CADMUS Jr | multiple countries | 1 | Y |
| Puig 2021^112^ | PLANETA | multiple countries | 1 | N |
| Rabe 2018^113^ | LIBERTY ASTHMA VENTURE | multiple countries | 2 | Y |
| Reich 2011^114^ | NCT00679731 | multiple countries | 1 | N |
| Reich 2017^115^ | VOYAGE 2 | multiple countries | 1 | Y |
| Reich 2019^116^ | ECLIPSE | multiple countries | 1 | Y |
| Reinisch 2011^117^ | ULTRA-1 | multiple countries | 1 | Y |
| Ryan 2018^118^ | IXORA-Q | multiple countries | 1 | N |
| Saeki 2015^119^ | UNCOVER-J | Japan | 1 | Y |
| Saini 2015^120^ | ASTERIA-1 | multiple countries | 1 | Y |
| Sandborn 2007^121^ | PRECISE-1 | multiple countries | 1 | Y |
| Sandborn 2012^122^ | ULTRA-2 | multiple countries | 1 | Y |
| Sandborn 2014^123^ | PURSUIT-M | multiple countries | 1 | N |
| Sandborn 2020^124^ | VISIBLE-1 | multiple countries | 1 | N |
| Sands 2019^125^ | UNIFI | multiple countries | 1 | Y |
| Sano 2018^126^ | NCT02343744 | Japan | 1 | N |
| Schreiber 2007^127^ | PRECISE-2 | multiple countries | 1 | Y |
| Sigurgeirsson 2021^128^ | ALLURE | multiple countries | 1 | N |
| Sigurgeirsson 2021^129^ | MATURE | multiple countries | 1 | N |
| Simpson 2020^130^ | LIBERTY AD ADOL | multiple countries | 2 | Y |
| Smolen 2009^131^ | GO-AFTER | multiple countries | 1 | Y |
| Smolen 2009^132^ | RAPID 2 | multiple countries | 3 | Y |
| Somerville 2014^133^ | NCT00500539 | multiple countries | 1 | Y |
| Stone 2017^134^ | GiACTA | multiple countries | 1 | Y |
| Sundy 2014^135^ | RESURGE | multiple countries | 2 | N |
| Takeuchi 2013^136^ | NCT00445770 | Japan | 1 | N |
| Takeuchi 2014^137^ | HOPEFUL 1 | Japan | 1 | Y |
| Tanaka 2011^138^ | GO-FORTH | Japan | 2 | Y |
| Tanida 2015^139^ | NCT01234671 | Japan | 1 | N |
| Taylor 2018^140^ | SIRROUND-H | multiple countries | 1 | Y |
| Terui 2019^141^ | NCT01845987 | Japan | 1 | N |
| Thaci 2020^142^ | POLARIS | Germany | 1 | Y |
| Tsai 2011^143^ | PEARL | multiple countries | 1 | Y |
| Tyring 2006^144^ | NCT00111449 | multiple countries | 1 | Y |
| Van der Heijde 2006^145^ | ATLAS | US | 1 | Y |
| Van der Heijde 2018^146^ | COAST-V | multiple countries | 1 | Y |
| Van De Putte 2004^147^ | none | multiple countries | 1 | Y |
| Vermeire 2021^148^ | VISIBLE-2 | multiple countries | 1 | N |
| Wei 2021^149^ | NCT02985983 | multiple countries | 1 | N |
| Weinblatt 2012^150^ | REALISTIC | multiple countries | 1 | Y |
| Weinblatt 2013^151^ | AMPLE | multiple countries | 3 | Y |
| Weinblatt 2017^152^ | NCT02167139 | multiple countries | 1 | Y |
| Wiland 2020^153^ | ADMYRA | multiple countries | 1 | Y |
| Yamamoto 2014^154^ | HIKARI | Japan | 3 | Y |
| Yamanaka 2020^155^ | 2015-002,809-12 | multiple countries | 1 | Y |
| Yamasaki 2017^156^ | NCT01782937 | Japan | 1 | N |
| Yamasaki 2020^157^ | none | Japan | 3 | N |
| Zhao 2021^158^ | NCT04345458 | China | 1 | Y |

References:

1. Alexeeva E., Horneff G., Dvoryakovskaya T., Denisova R., Nikishina I., Zholobova E., et al. Early combination therapy with etanercept and methotrexate in JIA patients shortens the time to reach an inactive disease state and remission: results of a double-blind placebo-controlled trial. Pediatr Rheumatol. 2021;19(1):5.

2. Atsumi T., Yamamoto K., Takeuchi T., Yamanaka H., Ishiguro N., Tanaka Y., et al. The first double-blind, randomised, parallel-group certolizumab pegol study in methotrexate-naive early rheumatoid arthritis patients with poor prognostic factors, C-OPERA, shows inhibition of radiographic progression. Ann Rheum Dis. 2016;75(1):75–83.

3. Bacharier L, Maspero J, Katelaris C, Fiocchi A, Gagnon R, de Mir I, et al. Dupilumab in Children with Uncontrolled Moderate-to-Severe Asthma. 2021;385(24):2230‐2240.

4. Bachelez H, van de Kerkhof P, Strohal R, Kubanov A, Valenzuela F, Lee J, et al. Tofacitinib versus etanercept or placebo in moderate-to-severe chronic plaque psoriasis: a phase 3 randomised non-inferiority trial. 2015;386(9993):552‐561.

5. Bachert C, Han J, Desrosiers M, Hellings P, Amin N, Lee S, et al. Efficacy and safety of dupilumab in patients with severe chronic rhinosinusitis with nasal polyps (LIBERTY NP SINUS-24 and LIBERTY NP SINUS-52): results from two multicentre, randomised, double-blind, placebo-controlled, parallel-group phase 3 trials. 2019;394(10209):1638‐1650.

6. Bao C, Huang F, Khan M, Fei K, Wu Z, Han C, et al. Safety and efficacy of golimumab in Chinese patients with active ankylosing spondylitis: 1-year results of a multicentre, randomized, double-blind, placebo-controlled phase III trial. 2014;53(9):1654‐1663.

7. Bi L., Li Y., He L., Xu H., Jiang Z., Wang Y., et al. Efficacy and safety of certolizumab pegol in combination with methotrexate in methotrexate-inadequate responder Chinese patients with active rheumatoid arthritis: 24-week results from a randomised, double-blind, placebo-controlled phase 3 study. Clin Exp Rheumatol. 2019;37(2):227–34.

8. Blauvelt A., Lacour J.-P., Fowler J.F., Weinberg J.M., Gospodinov D., Schuck E., et al. Phase III randomized study of the proposed adalimumab biosimilar GP2017 in psoriasis: impact of multiple switches. Br J Dermatol. 2018;179(3):623–31.

9. Bosch P.C. A randomized, double-blind, placebo controlled trial of adalimumab for interstitial cystitis/bladder pain syndrome. J Urol. 2014;191(1):77–82.

10. Brunner HI, Ruperto N, Tzaribachev N, Horneff G, Chasnyk VG, Panaviene V, et al. Subcutaneous golimumab for children with active polyarticular-course juvenile idiopathic arthritis: results of a multicentre, double-blind, randomised-withdrawal trial. Ann Rheum Dis. 2018 Jan;77(1):21–9.

11. Burmester G.R., Feist E., Kellner H., Braun J., Iking-Konert C., Rubbert-Roth A. Effectiveness and safety of the interleukin 6-receptor antagonist tocilizumab after 4 and 24 weeks in patients with active rheumatoid arthritis: The first phase IIIb real-life study (TAMARA). Ann Rheum Dis. 2011;70(5):755–9.

12. Burmester G.R., Lin Y., Patel R., Van Adelsberg J., Mangan E.K., Graham N.M.H., et al. Efficacy and safety of sarilumab monotherapy versus adalimumab monotherapy for the treatment of patients with active rheumatoid arthritis (MONARCH): A randomised, double-blind, parallel-group phase III trial. Ann Rheum Dis. 2017;76(5):840–7.

13. Callis Duffin K., Bagel J., Bukhalo M., Mercado Clement I.J., Choi S.L., Zhao F., et al. Phase 3, open-label, randomized study of the pharmacokinetics, efficacy and safety of ixekizumab following subcutaneous administration using a prefilled syringe or an autoinjector in patients with moderate-to-severe plaque psoriasis (UNCOVER-A). J Eur Acad Dermatol Venereol. 2017;31(1):107–13.

14. Castro M, Corren J, Pavord I, Maspero J, Wenzel S, Rabe K, et al. Dupilumab Efficacy and Safety in Moderate-to-Severe Uncontrolled Asthma. 2018;378(26):2486‐2496.

15. Chen X, Li Z, Wu H, Zhao D, Li X, Xu J, et al. A randomized, controlled trial of efficacy and safety of Anbainuo, a bio-similar etanercept, for moderate to severe rheumatoid arthritis inadequately responding to methotrexate. 2016;35(9):2175‐2183.

16. Chervinsky P, Casale T, Townley R, Tripathy I, Hedgecock S, Fowler-Taylor A, et al. Omalizumab, an anti-IgE antibody, in the treatment of adults and adolescents with perennial allergic rhinitis. 2003;91(2):160‐167.

17. Chupp G.L., Bradford E.S., Albers F.C., Bratton D.J., Wang-Jairaj J., Nelsen L.M., et al. Efficacy of mepolizumab add-on therapy on health-related quality of life and markers of asthma control in severe eosinophilic asthma (MUSCA): a randomised, double-blind, placebo-controlled, parallel-group, multicentre, phase 3b trial. Lancet Respir Med. 2017;5(5):390–400.

18. Coates L.C., Gossec L., Theander E., Bergmans P., Neuhold M., Karyekar C.S., et al. Efficacy and safety of guselkumab in patients with active psoriatic arthritis who are inadequate responders to tumour necrosis factor inhibitors: results through one year of a phase IIIb, randomised, controlled study (COSMOS). Ann Rheum Dis. 2021;((Coates) Nuffield Department of Orthopaedics, Rheumatology and Musculoskeletal Sciences, University of Oxford, Oxford, United Kingdom(Gossec) INSERM, Institut Pierre Louis d’Epidemiologie et de Sante Publique, Sorbonne Universite, Paris, France(Gossec) AP).

19. Coates LC, Pillai SG, Tahir H, Valter I, Chandran V, Kameda H, et al. Withdrawing Ixekizumab in Patients With Psoriatic Arthritis Who Achieved Minimal Disease Activity: Results From a Randomized, Double-Blind Withdrawal Study. Arthritis Rheumatol Hoboken NJ. 2021 Sep;73(9):1663–72.

20. Cohen S., Genovese M.C., Choy E., Perez-Ruiz F., Matsumoto A., Pavelka K., et al. Efficacy and safety of the biosimilar ABP 501 compared with adalimumab in patients with moderate to severe rheumatoid arthritis: A randomised, double-blind, phase III equivalence study. Ann Rheum Dis. 2017;76(10):1679–87.

21. Cohen S, Samad A, Karis E, Stolshek B, Trivedi M, Zhang H, et al. Decreased Injection Site Pain Associated with Phosphate-Free Etanercept Formulation in Rheumatoid Arthritis or Psoriatic Arthritis Patients: a Randomized Controlled Trial. 2019;6(2):245‐254.

22. Colombel JF, Sandborn WJ, Rutgeerts P, Enns R, Hanauer SB, Panaccione R, et al. Adalimumab for maintenance of clinical response and remission in patients with Crohn’s disease: the CHARM trial. Gastroenterology. 2007 Jan;132(1):52–65.

23. Colombel J.-F., Panaccione R., Bossuyt P., Lukas M., Baert F., Vanasek T., et al. Effect of tight control management on Crohn’s disease (CALM): a multicentre, randomised, controlled phase 3 trial. The Lancet. 2017;390(10114):2779–89.

24. D’Agostino M.A., Schett G., Lopez-Rdz A., Senolt L., Fazekas K., Burgos-Vargas R., et al. Response to Secukinumab on Synovitis using Power Doppler Ultrasound in Psoriatic Arthritis: 12-week Results from a Phase III Study, ULTIMATE. Rheumatol Oxf Engl. 2021;((D’Agostino) Department of Rheumatology, Catholic University of Sacred Heart, Roma, Italy(Schett) Department of Internal Medicine 3, Friedrich Alexander University of Erlangen-Nuremberg and Universitatsklinikum Erlangen, Erlangen, Germany(Schett) Deutsche).

25. De Benedetti F, Gattorno M, Anton J, Ben-Chetrit E, Frenkel J, Hoffman H, et al. Canakinumab for the Treatment of Autoinflammatory Recurrent Fever Syndromes. 2018;378(20):1908‐1919.

26. de Bruin-Weller M, Thaci D, Smith C, Reich K, Cork M, Radin A, et al. Dupilumab with concomitant topical corticosteroid treatment in adults with atopic dermatitis with an inadequate response or intolerance to ciclosporin A or when this treatment is medically inadvisable: a placebo-controlled, randomized phase III clinical. 2018;178(5):1083‐1101.

27. Deodhar A., Poddubnyy D., Pacheco-Tena C., Salvarani C., Lespessailles E., Rahman P., et al. Efficacy and Safety of Ixekizumab in the Treatment of Radiographic Axial Spondyloarthritis: Sixteen-Week Results From a Phase III Randomized, Double-Blind, Placebo-Controlled Trial in Patients With Prior Inadequate Response to or Intolerance of Tumor Necr. Arthritis Rheumatol. 2019;71(4):599–611.

28. Deodhar A., Helliwell P.S., Boehncke W.-H., Kollmeier A.P., Hsia E.C., Subramanian R.A., et al. Guselkumab in patients with active psoriatic arthritis who were biologic-naive or had previously received TNFalpha inhibitor treatment (DISCOVER-1): a double-blind, randomised, placebo-controlled phase 3 trial. The Lancet. 2020;395(10230):1115–25.

29. D’Haens G. Risk and benefits of biologic therapy for inflammatory bowel diseases. Gut. 2007;56(5):725–32.

30. Edwards C.J., Monnet J., Ullmann M., Vlachos P., Chyrok V., Ghori V. Safety of adalimumab biosimilar MSB11022 (acetate-buffered formulation) in patients with moderately-to-severely active rheumatoid arthritis. Clin Rheumatol. 2019;38(12):3381–90.

31. Elewski B.E., Okun M.M., Papp K., Baker C.S., Crowley J.J., Guillet G., et al. Adalimumab for nail psoriasis: Efficacy and safety from the first 26 weeks of a phase 3, randomized, placebo-controlled trial. J Am Acad Dermatol. 2018;78(1):90.

32. Emery P, Fleischmann R, Moreland L, Hsia E, Strusberg I, Durez P, et al. Golimumab, a human anti-tumor necrosis factor alpha monoclonal antibody, injected subcutaneously every four weeks in methotrexate-naive patients with active rheumatoid arthritis: twenty-four-week results of a phase III, multicenter, randomized, double-bl. 2009;60(8):2272‐2283.

33. Emery P, Vencovský J, Sylwestrzak A, Leszczyński P, Porawska W, Baranauskaite A, et al. A phase III randomised, double-blind, parallel-group study comparing SB4 with etanercept reference product in patients with active rheumatoid arthritis despite methotrexate therapy. 2017;76(1):51‐57.

34. Emery P, Rondon J, Parrino J, Lin Y, Pena-Rossi C, van Hoogstraten H, et al. Safety and tolerability of subcutaneous sarilumab and intravenous tocilizumab in patients with rheumatoid arthritis. 2019;58(5):849‐858.

35. Feldman S.R., Reznichenko N., Pulka G., Kingo K., GeorgeGaldava, Berti F., et al. Efficacy, Safety and Immunogenicity of AVT02 Versus Originator Adalimumab in Subjects with Moderate to Severe Chronic Plaque Psoriasis: A Multicentre, Double-Blind, Randomised, Parallel Group, Active Control, Phase III Study. BioDrugs. 2021;35(6):735–48.

36. Ferris L.K., Ott E., Jiang J., Hong H.C.-H., Li S., Han C., et al. Efficacy and safety of guselkumab, administered with a novel patient-controlled injector (One-Press), for moderate-to-severe psoriasis: results from the phase 3 ORION study. J Dermatol Treat. 2020;31(2):152–9.

37. Fleischmann R., Pangan A.L., Song I.-H., Mysler E., Bessette L., Peterfy C., et al. Upadacitinib Versus Placebo or Adalimumab in Patients With Rheumatoid Arthritis and an Inadequate Response to Methotrexate: Results of a Phase III, Double-Blind, Randomized Controlled Trial. Arthritis Rheumatol. 2019;71(11):1788–800.

38. Furst D.E., Kavanaugh A., Florentinus S., Kupper H., Karunaratne M., Birbara C.A. Final 10-year effectiveness and safety results from study DE020: Adalimumab treatment in patients with rheumatoid arthritis and an inadequate response to standard therapy. Rheumatol U K. 2015;54(12):2188–97.

39. Furst D.E., Shaikh S.A., Greenwald M., Bennett B., Davies O., Luijtens K., et al. Two dosing regimens of certolizumab pegol in patients with active rheumatoid arthritis. Arthritis Care Res. 2015;67(2):151–60.

40. Genovese MC, Fleischmann R, Kivitz AJ, Rell-Bakalarska M, Martincova R, Fiore S, et al. Sarilumab Plus Methotrexate in Patients With Active Rheumatoid Arthritis and Inadequate Response to Methotrexate: Results of a Phase III Study. Arthritis Rheumatol Hoboken NJ. 2015 Jun;67(6):1424–37.

41. Gevaert P, Omachi T, Corren J, Mullol J, Han J, Lee S, et al. Efficacy and safety of omalizumab in nasal polyposis: 2 randomized phase 3 trials. 2020;146(3):595‐605.

42. Gordon K, Blauvelt A, Papp K, Langley R, Luger T, Ohtsuki M, et al. Phase 3 Trials of Ixekizumab in Moderate-to-Severe Plaque Psoriasis. 2016;375(4):345‐356.

43. Gottlieb A, Langley R, Strober B, Papp K, Klekotka P, Creamer K, et al. A randomized, double-blind, placebo-controlled study to evaluate the addition of methotrexate to etanercept in patients with moderate to severe plaque psoriasis. 2012;167(3):649‐657.

44. Griffiths C.E.M., Strober B.E., Van De Kerkhof P., Ho V., Fidelus-Gort R., Yeilding N., et al. Comparison of ustekinumab and etanercept for moderate-to-severe psoriasis. N Engl J Med. 2010;362(2):118–28.

45. Hanania NA, Alpan O, Hamilos DL, Condemi JJ, Reyes-Rivera I, Zhu J, et al. Omalizumab in severe allergic asthma inadequately controlled with standard therapy: a randomized trial. Ann Intern Med. 2011 May 3;154(9):573–82.

46. Hercogova J., Papp K.A., Chyrok V., Ullmann M., Vlachos P., Edwards C.J. AURIEL-PsO: a randomized, double-blind phase III equivalence trial to demonstrate the clinical similarity of the proposed biosimilar MSB11022 to reference adalimumab in patients with moderate-to-severe chronic plaque-type psoriasis. Br J Dermatol. 2020;182(2):316–26.

47. Hibi T, Imai Y, Senoo A, Ohta K, Ukyo Y. Efficacy and safety of golimumab 52-week maintenance therapy in Japanese patients with moderate to severely active ulcerative colitis: a phase 3, double-blind, randomized, placebo-controlled study-(PURSUIT-J study). 2017;52(10):1101‐1111.

48. Horneff G, Burgos-Vargas R, Constantin T, Foeldvari I, Vojinovic J, Chasnyk V, et al. Efficacy and safety of open-label etanercept on extended oligoarticular juvenile idiopathic arthritis, enthesitis-related arthritis and psoriatic arthritis: part 1 (week 12) of the CLIPPER study. 2014;73(6):1114‐1122.

49. Hyams J, Griffiths A, Markowitz J, Baldassano R, Faubion W, Colletti R, et al. Safety and efficacy of adalimumab for moderate to severe Crohn’s disease in children. 2012;143(2):365‐74.e2.

50. Imagawa T., Nishikomori R., Takada H., Takeshita S., Patel N., Kim D., et al. Safety and efficacy of canakinumab in Japanese patients with phenotypes of cryopyrin-associated periodic syndrome as established in the first open-label, phase-3 pivotal study (24-week results). Clin Exp Rheumatol. 2013;31(2):0302–9.

51. Inman R, Davis J, Heijde D, Diekman L, Sieper J, Kim S, et al. Efficacy and safety of golimumab in patients with ankylosing spondylitis: results of a randomized, double-blind, placebo-controlled, phase III trial. 2008;58(11):3402‐3412.

52. Jaffe G.J., Dick A.D., Brezin A.P., Nguyen Q.D., Thorne J.E., Kestelyn P., et al. Adalimumab in patients with active noninfectious uveitis. N Engl J Med. 2016;375(10):932–43.

53. Kavanaugh A, McInnes I, Mease P, Krueger G, Gladman D, Gomez-Reino J, et al. Golimumab, a new human tumor necrosis factor alpha antibody, administered every four weeks as a subcutaneous injection in psoriatic arthritis: twenty-four-week efficacy and safety results of a randomized, placebo-controlled study. 2009;60(4):976‐986.

54. Kay J., Jaworski J., Wojciechowski R., Wiland P., Dudek A., Krogulec M., et al. Efficacy and safety of biosimilar CT-P17 versus reference adalimumab in subjects with rheumatoid arthritis: 24-week results from a randomized study. Arthritis Res Ther. 2021;23(1):51.

55. Keystone E., Van Der Heijde D., Mason Jr. D., Landewe R., Van Vollenhoven R., Combe B., et al. Certolizumab pegol plus methotrexate is significantly more effective than placebo plus methotrexate in active rheumatoid arthritis: Findings of a fifty-two-week, phase III, multicenter, randomized, double-blind, placebo-controlled, parallel-group study. Arthritis Rheum. 2008;58(11):3319–29.

56. Keystone E, Genovese M, Klareskog L, Hsia E, Hall S, Miranda P, et al. Golimumab, a human antibody to tumour necrosis factor {alpha} given by monthly subcutaneous injections, in active rheumatoid arthritis despite methotrexate therapy: the GO-FORWARD Study. 2009;68(6):789‐796.

57. Khanna D., Lin C.J.F., Goldin J., Kim G., Kuwana M., Allanore Y., et al. Tocilizumab in systemic sclerosis: a randomised, double-blind, placebo-controlled, phase 3 trial. Lancet Respir Med. 2020;8(10):963–74.

58. Kingsbury DJ, Bader-Meunier B, Patel G, Arora V, Kalabic J, Kupper H. Safety, effectiveness, and pharmacokinetics of adalimumab in children with polyarticular juvenile idiopathic arthritis aged 2 to 4 years. Clin Rheumatol. 2014;33(10):1433–41.

59. Kivitz A, Olech E, Borofsky M, Zazueta B, Navarro-Sarabia F, Radominski S, et al. Subcutaneous tocilizumab versus placebo in combination with disease-modifying antirheumatic drugs in patients with rheumatoid arthritis. 2014;66(11):1653‐1661.

60. Kobayashi S., Harigai M., Mozaffarian N., Pangan A.L., Sharma S., Brown L.S., et al. A multicenter, open-label, efficacy, pharmacokinetic, and safety study of adalimumab in Japanese patients with ankylosing spondylitis. Mod Rheumatol. 2012;22(4):589–97.

61. Kristensen L.E., Keiserman M., Papp K., McCasland L., White D., Lu W., et al. Efficacy and safety of risankizumab for active psoriatic arthritis: 24-week results from the randomised, double-blind, phase 3 KEEPsAKE 1 trial. Ann Rheum Dis. 2022;81(2):225–31.

62. Kuehr J, Brauburger J, Zielen S, Schauer U, Kamin W, Von Berg A, et al. Efficacy of combination treatment with anti-IgE plus specific immunotherapy in polysensitized children and adolescents with seasonal allergic rhinitis. J Allergy Clin Immunol. 2002 Feb;109(2):274–80.

63. Kuemmerle-Deschner J, Hachulla E, Cartwright R, Hawkins P, Tran T, Bader-Meunier B, et al. Two-year results from an open-label, multicentre, phase III study evaluating the safety and efficacy of canakinumab in patients with cryopyrin-associated periodic syndrome across different severity phenotypes. 2011;70(12):2095‐2102.

64. Landells I., Marano C., Hsu M.-C., Li S., Zhu Y., Eichenfield L.F., et al. Ustekinumab in adolescent patients age 12 to 17 years with moderate-to-severe plaque psoriasis: Results of the randomized phase 3 CADMUS study. J Am Acad Dermatol. 2015;73(4):594–603.

65. Landewe R., Braun J., Deodhar A., Dougados M., Maksymowych W.P., Mease P.J., et al. Efficacy of certolizumab pegol on signs and symptoms of axial spondyloarthritis including ankylosing spondylitis: 24-week results of a double-blind randomised placebo-controlled Phase 3 study. Ann Rheum Dis. 2014;73(1):39–47.

66. Langley R.G., Papp K., Gooderham M., Zhang L., Mallinckrodt C., Agada N., et al. Efficacy and safety of continuous every-2-week dosing of ixekizumab over 52 weeks in patients with moderate-to-severe plaque psoriasis in a randomized phase III trial (IXORA-P). Br J Dermatol. 2018;178(6):1315–23.

67. Langley R.G., Tsai T.-F., Flavin S., Song M., Randazzo B., Wasfi Y., et al. Efficacy and safety of guselkumab in patients with psoriasis who have an inadequate response to ustekinumab: results of the randomized, double-blind, phase III NAVIGATE trial. Br J Dermatol. 2018;178(1):114–23.

68. Lebwohl M., Strober B., Menter A., Gordon K., Weglowska J., Puig L., et al. Phase 3 studies comparing brodalumab with ustekinumab in psoriasis. N Engl J Med. 2015;373(14):1318–28.

69. Leonardi C, Powers J, Matheson R, Goffe B, Zitnik R, Wang A, et al. Etanercept as monotherapy in patients with psoriasis. 2003;349(21):2014‐2022.

70. Li Z, Zhang F, Kay J, Fei K, Han C, Zhuang Y, et al. Efficacy and safety results from a Phase 3, randomized, placebo-controlled trial of subcutaneous golimumab in Chinese patients with active rheumatoid arthritis despite methotrexate therapy. 2016;19(11):1143‐1156.

71. Lichtiger S., Binion D.G., Wolf D.C., Present D.H., Bensimon A.G., Wu E., et al. The CHOICE trial: Adalimumab demonstrates safety, fistula healing, improved quality of life and increased work productivity in patients with Crohns disease who failed prior infliximab therapy. Aliment Pharmacol Ther. 2010;32(10):1228–39.

72. Lofberg R., Louis E.V., Reinisch W., Robinson A.M., Kron M., Camez A., et al. Adalimumab produces clinical remission and reduces extraintestinal manifestations in Crohn’s disease: Results from CARE. Inflamm Bowel Dis. 2012;18(1):1–9.

73. Magnolo N., Kingo K., Laquer V., Browning J., Reich A., Szepietowski J.C., et al. A phase 3 open-label, randomized multicenter study to evaluate efficacy and safety of secukinumab in pediatric patients with moderate to severe plaque psoriasis: 24-week results. J Am Acad Dermatol. 2022;86(1):122–30.

74. Matsuno H, Tomomitsu M, Hagino A, Shin S, Lee J, Song Y. Phase III, multicentre, double-blind, randomised, parallel-group study to evaluate the similarities between LBEC0101 and etanercept reference product in terms of efficacy and safety in patients with active rheumatoid arthritis inadequately responding to. 2018;77(4):488‐494.

75. Matucci-Cerinic M., Allanore Y., Kavanaugh A., Buch M.H., Schulze-Koops H., Kucharz E.J., et al. Efficacy, safety and immunogenicity of GP2015, an etanercept biosimilar, compared with the reference etanercept in patients with moderate-To-severe rheumatoid arthritis: 24-week results from the comparative phase III, randomised, double-blind EQUIRA study. RMD Open. 2018;4(2):e000757.

76. McInnes I.B., Kavanaugh A., Gottlieb A.B., Puig L., Rahman P., Ritchlin C., et al. Efficacy and safety of ustekinumab in patients with active psoriatic arthritis: 1 year results of the phase 3, multicentre, double-blind, placebo-controlled PSUMMIT 1 trial. The Lancet. 2013;382(9894):780–9.

77. McInnes I.B., Behrens F., Mease P.J., Kavanaugh A., Ritchlin C., Nash P., et al. Secukinumab versus adalimumab for treatment of active psoriatic arthritis (EXCEED): a double-blind, parallel-group, randomised, active-controlled, phase 3b trial. Lancet Lond Engl. 2020;395(10235):1496–505.

78. Mease P.J., Fleischmann R., Deodhar A.A., Wollenhaupt J., Khraishi M., Kielar D., et al. Effect of certolizumab pegol on signs and symptoms in patients with psoriatic arthritis: 24-week results of a Phase 3 double-blind randomised placebo-controlled study (RAPID-PsA). Ann Rheum Dis. 2014;73(1):48–55.

79. Mease PJ, van der Heijde D, Ritchlin CT, Okada M, Cuchacovich RS, Shuler CL, et al. Ixekizumab, an interleukin-17A specific monoclonal antibody, for the treatment of biologic-naive patients with active psoriatic arthritis: results from the 24-week randomised, double-blind, placebo-controlled and active (adalimumab)-controlled period of t. Ann Rheum Dis. 2017;76(1):79–87.

80. Mease P, Gladman D, Collier D, Ritchlin C, Helliwell P, Liu L, et al. Etanercept and Methotrexate as Monotherapy or in Combination for Psoriatic Arthritis: primary Results From a Randomized, Controlled Phase III Trial. 2019;71(7):1112‐1124.

81. Mease P, Smolen J, Behrens F, Nash P, Liu-Leage S, Li L, et al. A head-to-head comparison of the efficacy and safety of ixekizumab and adalimumab in biological-naïve patients with active psoriatic arthritis: 24-week results of a randomised, open-label, blinded-assessor trial. Ann Rheum Dis. 2019 Sep 28;79:annrheumdis-2019.

82. Mease P.J., Rahman P., Gottlieb A.B., Kollmeier A.P., Hsia E.C., Xu X.L., et al. Guselkumab in biologic-naive patients with active psoriatic arthritis (DISCOVER-2): a double-blind, randomised, placebo-controlled phase 3 trial. The Lancet. 2020;395(10230):1126–36.

83. Menter A., Tyring S.K., Gordon K., Kimball A.B., Leonardi C.L., Langley R.G., et al. Adalimumab therapy for moderate to severe psoriasis: A randomized, controlled phase III trial. J Am Acad Dermatol. 2008;58(1):106–15.

84. Menter A., Arenberger P., Balser S., Beissert S., Cauthen A., Czeloth N., et al. Similar efficacy, safety, and immunogenicity of the biosimilar BI 695501 and adalimumab reference product in patients with moderate-to-severe chronic plaque psoriasis: results from the randomized Phase III VOLTAIRE-PSO study. Expert Opin Biol Ther. 2021;21(1):87–96.

85. Menzies-Gow A, Corren J, Bourdin A, Chupp G, Israel E, Wechsler ME, et al. Tezepelumab in Adults and Adolescents with Severe, Uncontrolled Asthma. N Engl J Med. 2021 May 13;384(19):1800–9.

86. Miyasaka N. Clinical investigation in highly disease-affected rheumatoid arthritis patients in Japan with adalimumab applying standard and general evaluation: The CHANGE study. Mod Rheumatol. 2008;18(3):252–62.

87. Mitha E., Ralph Schumacher H., Fouche L., Luo S.-F., Weinstein S.P., Yancopoulos G.D., et al. Rilonacept for gout flare prevention during initiation of uric acid-lowering therapy: Results from the PRESURGE-2 international, phase 3, randomized, placebo-controlled trial. Rheumatol U K. 2013;52(7):1285–92.

88. Moreland L, Schiff M, Baumgartner S, Tindall E, Fleischmann R, Bulpitt K, et al. Etanercept therapy in rheumatoid arthritis. A randomized, controlled trial. 1999;130(6):478‐486.

89. Morita A., Yamazaki F., Matsuyama T., Takahashi K., Arai S., Asahina A., et al. Adalimumab treatment in Japanese patients with generalized pustular psoriasis: Results of an open-label phase 3 study. J Dermatol. 2018;45(12):1371–80.

90. Morita A., Takahashi H., Ozawa K., Imafuku S., Nakama T., Takahashi K., et al. Twenty-four-week interim analysis from a phase 3 open-label trial of adalimumab in Japanese patients with moderate to severe hidradenitis suppurativa. J Dermatol. 2019;46(9):745–51.

91. Motoya S, Watanabe K, Ogata H, Kanai T, Matsui T, Suzuki Y, et al. Vedolizumab in Japanese patients with ulcerative colitis: A Phase 3, randomized, double-blind, placebo-controlled study. Green J, editor. PLOS ONE. 2019 Feb 26;14(2):e0212989.

92. Muller-Ladner U, Flipo R, Vincendon P, Brault Y, Kielar D. Comparison of patient satisfaction with two different etanercept delivery systems: a randomised controlled study in patients with rheumatoid arthritis. 2012;71(10):890‐899.

93. Mysler E., Cardiel M.H., Xavier R.M., Lopez A., Ramos-Esquivel A. Subcutaneous Tocilizumab in Monotherapy or in Combination With Nonbiologic Disease-Modifying Antirheumatic Drugs in Latin American Patients With Moderate to Severe Active Rheumatoid Arthritis: A Multicenter, Phase IIIb Study. J Clin Rheumatol Pract Rep Rheum Musculoskelet Dis. 2020;26(7S Suppl 2):S180–6.

94. Kirkham B., Okada M., Rahman P., Adams D.H., Kerr L., Lee C., et al. Ixekizumab for the treatment of patients with active psoriatic arthritis and an inadequate response to tumour necrosis factor inhibitors: results from the 24-week randomised, double-blind, placebo-controlled period of the SPIRIT-P2 phase 3 trial. The Lancet. 2017;389(10086):2317–27.

95. Nash P., Mease P.J., McInnes I.B., Rahman P., Ritchlin C.T., Blanco R., et al. Efficacy and safety of secukinumab administration by autoinjector in patients with psoriatic arthritis: Results from a randomized, placebo-controlled trial (FUTURE 3). Arthritis Res Ther. 2018;20(1):47.

96. Nishimura K, Hara R, Umebayashi H, Takei S, Iwata N, Imagawa T, et al. Efficacy and safety of canakinumab in systemic juvenile idiopathic arthritis: 48-week results from an open-label phase III study in Japanese patients. Mod Rheumatol. 2021;31(1):226–34.

97. Ogata A, Tanimura K, Sugimoto T, Inoue H, Urata Y, Matsubara T, et al. Phase III study of the efficacy and safety of subcutaneous versus intravenous tocilizumab monotherapy in patients with rheumatoid arthritis. 2014;66(3):344‐354.

98. Ohtsuki M, Kubo H, Morishima H, Goto R, Zheng R, Nakagawa H. Guselkumab, an anti-interleukin-23 monoclonal antibody, for the treatment of moderate to severe plaque-type psoriasis in Japanese patients: efficacy and safety results from a phase 3, randomized, double-blind, placebo-controlled study. 2018;45(9):1053‐1062.

99. Ostor A., Van den Bosch F., Papp K., Asnal C., Blanco R., Aelion J., et al. Efficacy and safety of risankizumab for active psoriatic arthritis: 24-week results from the randomised, double-blind, phase 3 KEEPsAKE 2 trial. Ann Rheum Dis. 2021;((Ostor) Monash Medical School, Cabrini Hospital and Emertius Research, Melbourne, VIC, Australia(Van den Bosch) Department of Rheumatology, Ghent University, VIB Center for Inflammation Research, Gent, Belgium(Papp) Probity Medical Research-K Papp Clinica).

100. Paller A, Siegfried E, Langley R, Gottlieb A, Pariser D, Landells I, et al. Etanercept treatment for children and adolescents with plaque psoriasis. 2008;358(3):241‐251.

101. Paller A, Seyger M, Alejandro Magariños G, Bagel J, Pinter A, Cather J, et al. Efficacy and safety of ixekizumab in a phase III, randomized, double-blind, placebo-controlled study in paediatric patients with moderate-to-severe plaque psoriasis (IXORA-PEDS). 2020;183(2):231‐241.

102. Paller AS, Siegfried EC, Thaçi D, Wollenberg A, Cork MJ, Arkwright PD, et al. Efficacy and safety of dupilumab with concomitant topical corticosteroids in children 6 to 11 years old with severe atopic dermatitis: A randomized, double-blinded, placebo-controlled phase 3 trial. J Am Acad Dermatol. 2020 Jun;S019096222031152X.

103. Panaccione R., Loftus Jr. E.V., Binion D., McHugh K., Alam S., Chen N., et al. Efficacy and safety of adalimumab in Canadian patients with moderate to severe Crohn’s disease: Results of the adalimumab in Canadian subjects with moderate to severe Crohn’s diseaSe (ACCESS) trial. Can J Gastroenterol. 2011;25(8):419–25.

104. Papp K, Tyring S, Lahfa M, Prinz J, Griffiths C, Nakanishi A, et al. A global phase III randomized controlled trial of etanercept in psoriasis: safety, efficacy, and effect of dose reduction. 2005;152(6):1304‐1312.

105. Papp KA, Langley RG, Lebwohl M, Krueger GG, Szapary P, Yeilding N, et al. Efficacy and safety of ustekinumab, a human interleukin-12/23 monoclonal antibody, in patients with psoriasis: 52-week results from a randomised, double-blind, placebo-controlled trial (PHOENIX 2). Lancet Lond Engl. 2008 May 17;371(9625):1675–84.

106. Papp K, Barber K, Bissonnette R, Bourcier M, Lynde C, Poulin Y, et al. A Randomized, blinded assessor study to Evaluate the efFIcacy and safety of etanercept 50 mg once weekly plus as Needed topical agent vs. Etanercept 50 mg twice weekly in patients with moderate to severe plaque psoriasis (REFINE). 2015;29(2):361‐366.

107. Papp K.A., Reich K., Paul C., Blauvelt A., Baran W., Bolduc C., et al. A prospective phase III, randomized, double-blind, placebo-controlled study of brodalumab in patients with moderate-to-severe plaque psoriasis. Br J Dermatol [Internet]. 2016;((Papp) Probity Medical Research and K Papp Clinical Research Waterloo, ON Canada(Reich) Dermatologikum Hamburg and SCIderm Research Institute Hamburg Germany(Paul) Paul Sabatier University Toulouse France(Blauvelt) Oregon Medical Research Center Portland,). Available from: http://www.blackwellpublishing.com/journals/BJD

108. Papp K, Thaci D, Marcoux D, Weibel L, Philipp S, Ghislain PD, et al. Efficacy and safety of adalimumab every other week versus methotrexate once weekly in children and adolescents with severe chronic plaque psoriasis: a randomised, double-blind, phase 3 trial. 2017;(no pagination). Available from: https://www.cochranelibrary.com/central/doi/10.1002/central/CN-01374450/full

109. Papp K, Bachelez H, Costanzo A, Foley P, Gooderham M, Kaur P, et al. Clinical similarity of the biosimilar ABP 501 compared with adalimumab after single transition: long-term results from a randomized controlled, double-blind, 52-week, phase III trial in patients with moderate-to-severe plaque psoriasis. Br J Dermatol. 2017 Dec;177(6):1562–74.

110. Paul C., Lacour J.-P., Tedremets L., Kreutzer K., Jazayeri S., Adams S., et al. Efficacy, safety and usability of secukinumab administration by autoinjector/pen in psoriasis: A randomized, controlled trial (JUNCTURE). J Eur Acad Dermatol Venereol. 2015;29(6):1082–90.

111. Philipp S., Menter A., Nikkels A.F., Barber K., Landells I., Eichenfield L.F., et al. Ustekinumab for the treatment of moderate-to-severe plaque psoriasis in paediatric patients (>= 6 to < 12 years of age): efficacy, safety, pharmacokinetic and biomarker results from the open-label CADMUS Jr study. Br J Dermatol. 2020;183(4):664–72.

112. Puig L., Bakulev A.L., Kokhan M.M., Samtsov A.V., Khairutdinov V.R., Morozova M.A., et al. Efficacy and Safety of Netakimab, A Novel Anti-IL-17 Monoclonal Antibody, in Patients with Moderate to Severe Plaque Psoriasis. Results of A 54-Week Randomized Double-Blind Placebo-Controlled PLANETA Clinical Trial. Dermatol Ther. 2021;11(4):1319–32.

113. Rabe K, Nair P, Brusselle G, Maspero J, Castro M, Sher L, et al. Efficacy and Safety of Dupilumab in Glucocorticoid-Dependent Severe Asthma. 2018;378(26):2475‐2485.

114. Reich K., Langley R.G., Papp K.A., Ortonne J.-P., Unnebrink K., Kaul M., et al. A 52-week trial comparing briakinumab with methotrexate in patients with psoriasis. N Engl J Med. 2011;365(17):1586–96.

115. Reich K., Armstrong A.W., Foley P., Song M., Wasfi Y., Randazzo B., et al. Efficacy and safety of guselkumab, an anti-interleukin-23 monoclonal antibody, compared with adalimumab for the treatment of patients with moderate to severe psoriasis with randomized withdrawal and retreatment: Results from the phase III, double-blind, p. J Am Acad Dermatol. 2017;76(3):418–31.

116. Reich K., Armstrong A.W., Langley R.G., Flavin S., Randazzo B., Li S., et al. Guselkumab versus secukinumab for the treatment of moderate-to-severe psoriasis (ECLIPSE): results from a phase 3, randomised controlled trial. The Lancet. 2019;394(10201):831–9.

117. Reinisch W., Sandborn W.J., Hommes D.W., D’Haens G., Hanauer S., Schreiber S., et al. Adalimumab for induction of clinical remission in moderately to severely active ulcerative colitis: Results of a randomised controlled trial. Gut. 2011;60(6):780–7.

118. Ryan C, Menter A, Guenther L, Blauvelt A, Bissonnette R, Meeuwis K, et al. Efficacy and safety of ixekizumab in a randomized, double-blinded, placebo-controlled phase IIIb study of patients with moderate-to-severe genital psoriasis. Br J Dermatol. 2018 Oct;179(4):844–52.

119. Saeki H., Nakagawa H., Ishii T., Morisaki Y., Aoki T., Berclaz P.-Y., et al. Efficacy and safety of open-label ixekizumab treatment in Japanese patients with moderate-to-severe plaque psoriasis, erythrodermic psoriasis and generalized pustular psoriasis. J Eur Acad Dermatol Venereol. 2015;29(6):1148–55.

120. Saini S, Bindslev-Jensen C, Maurer M, Grob J, Bülbül Baskan E, Bradley M, et al. Efficacy and safety of omalizumab in patients with chronic idiopathic/spontaneous urticaria who remain symptomatic on H1 antihistamines: a randomized, placebo-controlled study. 2015;135(1):67‐75.

121. Sandborn WJ, Feagan BG, Stoinov S, Honiball PJ, Rutgeerts P, Mason D, et al. Certolizumab Pegol for the Treatment of Crohn’s Disease. N Engl J Med. 2007 Jul 19;357(3):228–38.

122. Sandborn W.J., Van Assche G., Reinisch W., Colombel J., D’Haens G., Wolf D.C., et al. Adalimumab induces and maintains clinical remission in patients with moderate-to-severe ulcerative colitis. Gastroenterology. 2012;142(2):257.

123. Sandborn W, Feagan B, Marano C, Zhang H, Strauss R, Johanns J, et al. Subcutaneous golimumab maintains clinical response in patients with moderate-to-severe ulcerative colitis. 2014;146(1):96‐109.e1.

124. Sandborn W, Baert F, Danese S, Krznarić Ž, Kobayashi T, Yao X, et al. Efficacy and Safety of Vedolizumab Subcutaneous Formulation in a Randomized Trial of Patients With Ulcerative Colitis. 2020;158(3):562‐572.e12.

125. Sands B.E., Sandborn W.J., Panaccione R., O’Brien C.D., Zhang H., Johanns J., et al. Ustekinumab as induction and maintenance therapy for ulcerative colitis. N Engl J Med. 2019;381(13):1201–14.

126. Sano S., Kubo H., Morishima H., Goto R., Zheng R., Nakagawa H. Guselkumab, a human interleukin-23 monoclonal antibody in Japanese patients with generalized pustular psoriasis and erythrodermic psoriasis: Efficacy and safety analyses of a 52-week, phase 3, multicenter, open-label study. J Dermatol. 2018;45(5):529–39.

127. Schreiber S, Khaliq-Kareemi M, Lawrance IC, Thomsen OØ, Hanauer SB, McColm J, et al. Maintenance therapy with certolizumab pegol for Crohn’s disease. N Engl J Med. 2007 Jul 19;357(3):239–50.

128. Sigurgeirsson B., Schakel K., Hong C.-H., Effendy I., Placek W., Rich P., et al. Efficacy, tolerability, patient usability, and satisfaction with a 2 mL pre-filled syringe containing secukinumab 300 mg in patients with moderate to severe plaque psoriasis: results from the phase 3 randomized, double-blind, placebo-controlled ALLURE stu. J Dermatol Treat [Internet]. 2021;((Sigurgeirsson) Department of Dermatology, Faculty of Medicine, University of Iceland, Reykjavik, Iceland(Schakel) Department of Dermatology, Heidelberg University Hospital, Heidelberg and Interdisciplinary Center of Chronic Inflammatory Diseases, Heidelb). Available from: http://www.tandfonline.com/loi/ijdt20

129. Sigurgeirsson B, Browning J, Tyring S, Szepietowski JC, Rivera-Díaz R, Effendy I, et al. Secukinumab demonstrates efficacy, safety, and tolerability upon administration by 2 ml autoinjector in adult patients with plaque psoriasis: 52-week results from MATURE, a randomized, placebo-controlled trial. Dermatol Ther. 2022;35(3):e15285.

130. Simpson EL, Paller AS, Siegfried EC, Boguniewicz M, Sher L, Gooderham MJ, et al. Efficacy and Safety of Dupilumab in Adolescents With Uncontrolled Moderate to Severe Atopic Dermatitis: A Phase 3 Randomized Clinical Trial. JAMA Dermatol. 2020 Jan 1;156(1):44.

131. Smolen J, Kay J, Doyle M, Landewé R, Matteson E, Wollenhaupt J, et al. Golimumab in patients with active rheumatoid arthritis after treatment with tumour necrosis factor alpha inhibitors (GO-AFTER study): a multicentre, randomised, double-blind, placebo-controlled, phase III trial. 2009;374(9685):210‐221.

132. Smolen J., Landewe R.B., Mease P., Brzezicki J., Mason D., Luijtens K., et al. Efficacy and safety of certolizumab pegol plus methotrexate in active rheumatoid arthritis: The RAPID 2 study. A randomised controlled trial. Ann Rheum Dis. 2009;68(6):797–804.

133. Somerville L., Bardelas J., Viegas A., D’andrea P., Blogg M., Peachey G. Immunogenicity and safety of omalizumab in pre-filled syringes in patients with allergic (IgE-mediated) asthma. Curr Med Res Opin. 2014;30(1):59–66.

134. Stone J, Tuckwell K, Dimonaco S, Klearman M, Aringer M, Blockmans D, et al. Trial of Tocilizumab in Giant-Cell Arteritis. 2017;377(4):317‐328.

135. Sundy JS, Schumacher HR, Kivitz A, Weinstein SP, Wu R, King-Davis S, et al. Rilonacept for gout flare prevention in patients receiving uric acid-lowering therapy: results of RESURGE, a phase III, international safety study. J Rheumatol. 2014 Aug;41(8):1703–11.

136. Takeuchi T, Miyasaka N, Zang C, Alvarez D, Fletcher T, Wajdula J, et al. A phase 3 randomized, double-blind, multicenter comparative study evaluating the effect of etanercept versus methotrexate on radiographic outcomes, disease activity, and safety in Japanese subjects with active rheumatoid arthritis. Mod Rheumatol. 2013 Jul;23(4):623–33.

137. Takeuchi T., Yamanaka H., Ishiguro N., Miyasaka N., Mukai M., Matsubara T., et al. Adalimumab, a human anti-TNF monoclonal antibody, outcome study for the prevention of joint damage in Japanese patients with early rheumatoid arthritis: The HOPEFUL 1 study. Ann Rheum Dis. 2014;73(3):536–43.

138. Tanaka Y, Harigai M, Takeuchi T, Yamanaka H, Ishiguro N, Yamamoto K, et al. Golimumab in combination with methotrexate in Japanese patients with active rheumatoid arthritis: results of the GO-FORTH study. 2012;71(6):817‐824.

139. Tanida S., Inoue N., Kobayashi K., Naganuma M., Hirai F., Iizuka B., et al. Adalimumab for the treatment of japanese patients with intestinal behcet’s disease. Clin Gastroenterol Hepatol. 2015;13(5):940–8.

140. Taylor P.C., Schiff M.H., Wang Q., Jiang Y., Zhuang Y., Kurrasch R., et al. Efficacy and safety of monotherapy with sirukumab compared with adalimumab monotherapy in biologic-naive patients with active rheumatoid arthritis (SIRROUND-H): A randomised, double-blind, parallel-group, multinational, 52-week, phase 3 study. Ann Rheum Dis. 2018;77(5):658–66.

141. Terui T, Kobayashi S, Okubo Y, Murakami M, Hirose K, Kubo H. Efficacy and Safety of Guselkumab, an Anti–interleukin 23 Monoclonal Antibody, for Palmoplantar Pustulosis: A Randomized Clinical Trial. JAMA Dermatol. 2018 Mar 1;154(3):309.

142. Thaci D., Pinter A., Sebastian M., Termeer C., Sticherling M., Gerdes S., et al. Guselkumab is superior to fumaric acid esters in patients with moderate-to-severe plaque psoriasis who are naive to systemic treatment: results from a randomized, active-comparator-controlled phase IIIb trial (POLARIS). Br J Dermatol. 2020;183(2):265–75.

143. Tsai TF, Ho JC, Song M, Szapary P, Guzzo C, Shen YK, et al. Efficacy and safety of ustekinumab for the treatment of moderate-to-severe psoriasis: A phase III, randomized, placebo-controlled trial in Taiwanese and Korean patients (PEARL). J Dermatol Sci. 2011 Sep;63(3):154–63.

144. Tyring S, Gottlieb A, Papp K, Gordon K, Leonardi C, Wang A, et al. Etanercept and clinical outcomes, fatigue, and depression in psoriasis: double-blind placebo-controlled randomised phase III trial. 2006;367(9504):29‐35.

145. van der Heijde D, Kivitz A, Schiff MH, Sieper J, Dijkmans BAC, Braun J, et al. Efficacy and safety of adalimumab in patients with ankylosing spondylitis: results of a multicenter, randomized, double-blind, placebo-controlled trial. Arthritis Rheum. 2006 Jul;54(7):2136–46.

146. van der Heijde D., Cheng-Chung Wei J., Dougados M., Mease P., Deodhar A., Maksymowych W.P., et al. Ixekizumab, an interleukin-17A antagonist in the treatment of ankylosing spondylitis or radiographic axial spondyloarthritis in patients previously untreated with biological disease-modifying anti-rheumatic drugs (COAST-V): 16 week results of a phase 3 ra. The Lancet. 2018;392(10163):2441–51.

147. Van De Putte L.B.A., Atkins C., Malaise M., Sany J., Russell A.S., Van Riel P.L.C.M., et al. Efficacy and safety of adalimumab as monotherapy in patients with rheumatoid arthritis for whom previous disease modifying antirheumatic drug treatment has failed. Ann Rheum Dis. 2004;63(5):508–16.

148. Vermeire S, D’Haens G, Baert F, Danese S, Kobayashi T, Loftus EV, et al. Efficacy and Safety of Subcutaneous Vedolizumab in Patients With Moderately to Severely Active Crohn’s Disease: Results From the VISIBLE 2 Randomised Trial. J Crohns Colitis. 2022 Jan 28;16(1):27–38.

149. Wei JCC, Kim TH, Kishimoto M, Ogusu N, Jeong H, Kobayashi S. Efficacy and safety of brodalumab, an anti-IL17RA monoclonal antibody, in patients with axial spondyloarthritis: 16-week results from a randomised, placebo-controlled, phase 3 trial. Ann Rheum Dis. 2021 Aug 1;80(8):1014–21.

150. Weinblatt M.E., Fleischmann R., Huizinga T.W.J., Emery P., Pope J., Massarotti E.M., et al. Efficacy and safety of certolizumab pegol in a broad population of patients with active rheumatoid arthritis: Results from the REALISTIC phase IIIb study. Rheumatol U K. 2012;51(12):2204–14.

151. Weinblatt ME, Schiff M, Valente R, van der Heijde D, Citera G, Zhao C, et al. Head-to-head comparison of subcutaneous abatacept versus adalimumab for rheumatoid arthritis: findings of a phase IIIb, multinational, prospective, randomized study. Arthritis Rheum. 2013;65(1):28–38.

152. Weinblatt M.E., Baranauskaite A., Niebrzydowski J., Dokoupilova E., Zielinska A., Jaworski J., et al. Phase III Randomized Study of SB5, an Adalimumab Biosimilar, Versus Reference Adalimumab in Patients With Moderate-to-Severe Rheumatoid Arthritis. Arthritis Rheumatol. 2018;70(1):40–8.

153. Wiland P., Jeka S., Dokoupilova E., Brandt-Jurgens J., Miranda Limon J.M., Cantalejo Moreira M., et al. Switching to Biosimilar SDZ-ADL in Patients with Moderate-to-Severe Active Rheumatoid Arthritis: 48-Week Efficacy, Safety and Immunogenicity Results From the Phase III, Randomized, Double-Blind ADMYRA Study. BioDrugs. 2020;34(6):809–23.

154. Yamamoto K, Takeuchi T, Yamanaka H, Ishiguro N, Tanaka Y, Eguchi K, et al. Efficacy and safety of certolizumab pegol without methotrexate co-administration in Japanese patients with active rheumatoid arthritis: The HIKARI randomized, placebo-controlled trial. Mod Rheumatol. 2014 Jul 1;24(4):552–60.

155. Yamanaka H, Kamatani N, Tanaka Y, Hibino T, Drescher E, Sanchez-Burson J, et al. A Comparative Study to Assess the Efficacy, Safety, and Immunogenicity of YLB113 and the Etanercept Reference Product for the Treatment of Patients with Rheumatoid Arthritis. 2020;7(1):149‐163.

156. Yamasaki K., Nakagawa H., Kubo Y., Ootaki K. Efficacy and safety of brodalumab in patients with generalized pustular psoriasis and psoriatic erythroderma: results from a 52-week, open-label study. Br J Dermatol. 2017;176(3):741–51.

157. Yamasaki K., Yamanaka K., Zhao Y., Iwano S., Takei K., Suzuki K., et al. Adalimumab in Japanese patients with active ulcers of pyoderma gangrenosum: Twenty-six-week phase 3 open-label study. J Dermatol. 2020;47(12):1383–90.

158. Zhao D, He D, Bi L, Wu H, Liu Y, Wu Z, et al. Safety and Efficacy of Prefilled Liquid Etanercept-Biosimilar Yisaipu for Active Ankylosing Spondylitis: A Multi-Center Phase III Trial. Rheumatol Ther. 2021 Mar;8(1):361–74.
