# Supplementary material for: A Systematic Review and Meta-Analysis of Injection Site Reactions in Randomized-Controlled Trials of Biologic Injections
Source: J Cutan Med Surg. 2023 Aug 2;27(4):358–67. doi: 10.1177/12034754231188444 (PMC10486173; doi:10.1177/12034754231188444)
Supplement: Online supplementary file 4 - Supplemental material for A Systematic Review and Meta-Analysis of Injection Site Reactions in Randomized-Controlled Trials of Biologic Injections [file sj-docx-4-cms-10.1177_12034754231188444.docx]

1. Brodalumab


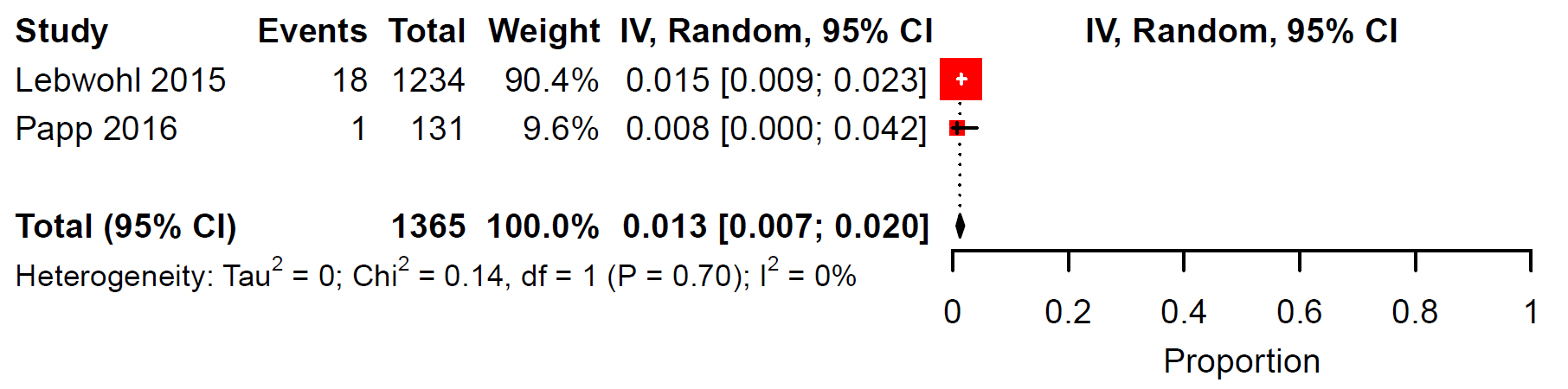


1. Canakinumab

**
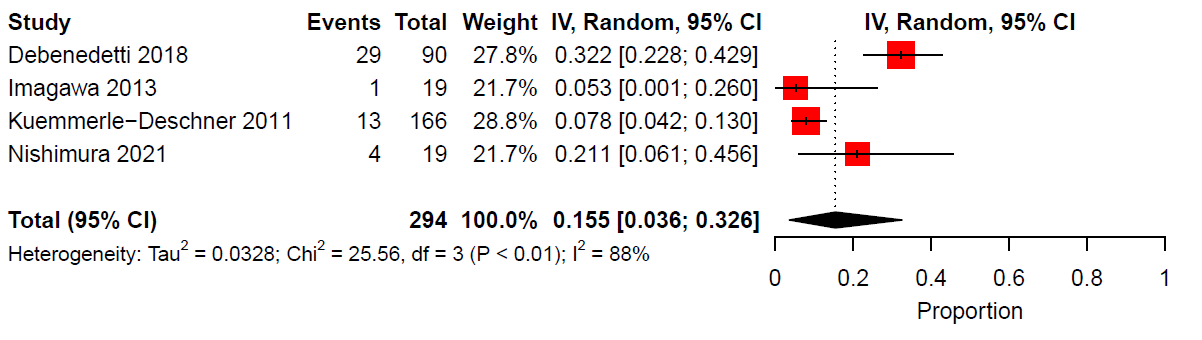
**

1. Dupilumab


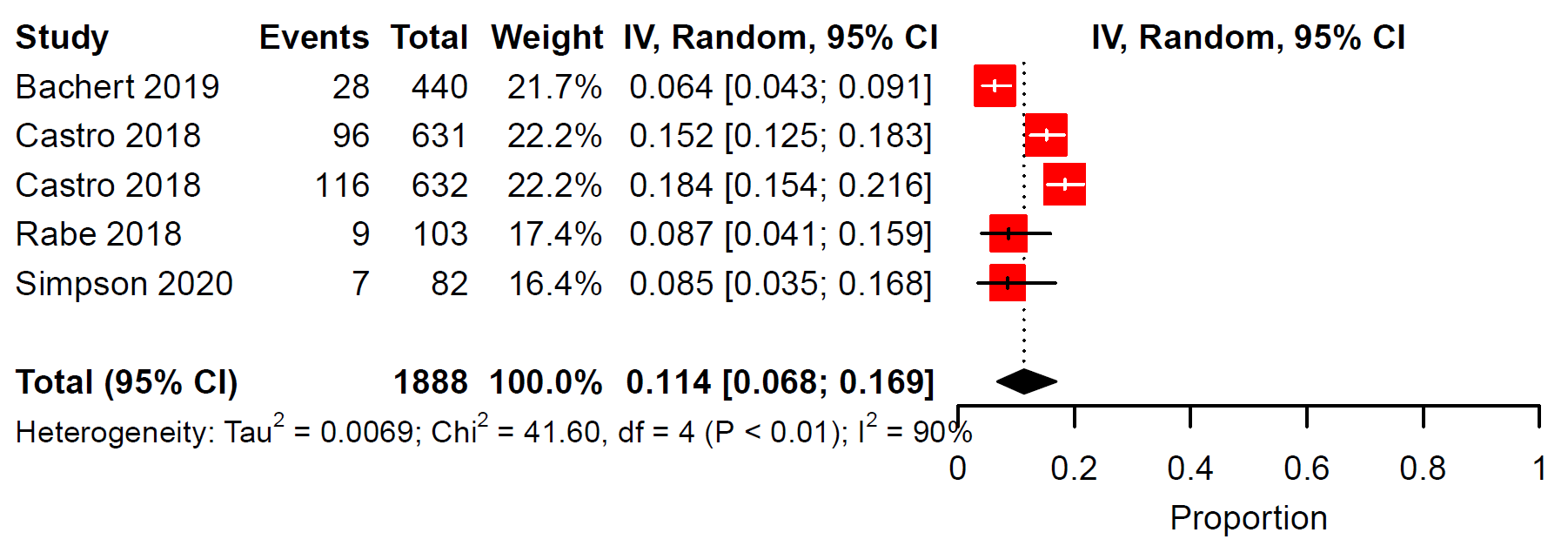


1. Golimumab


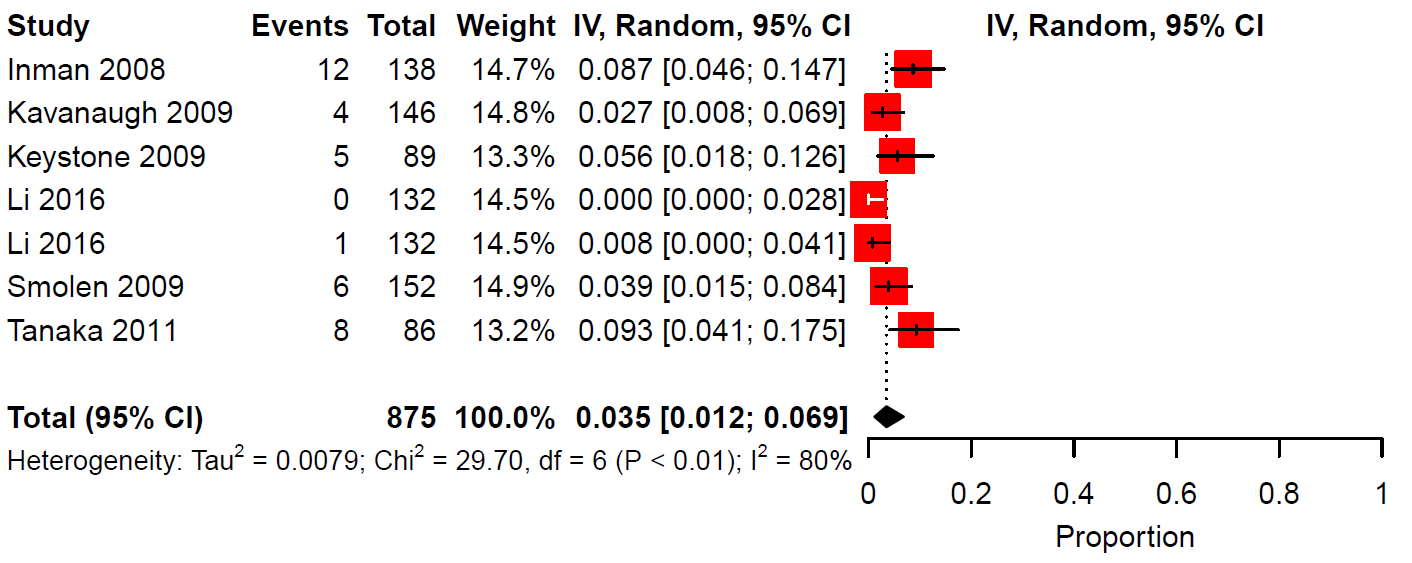


1. Guselkumab


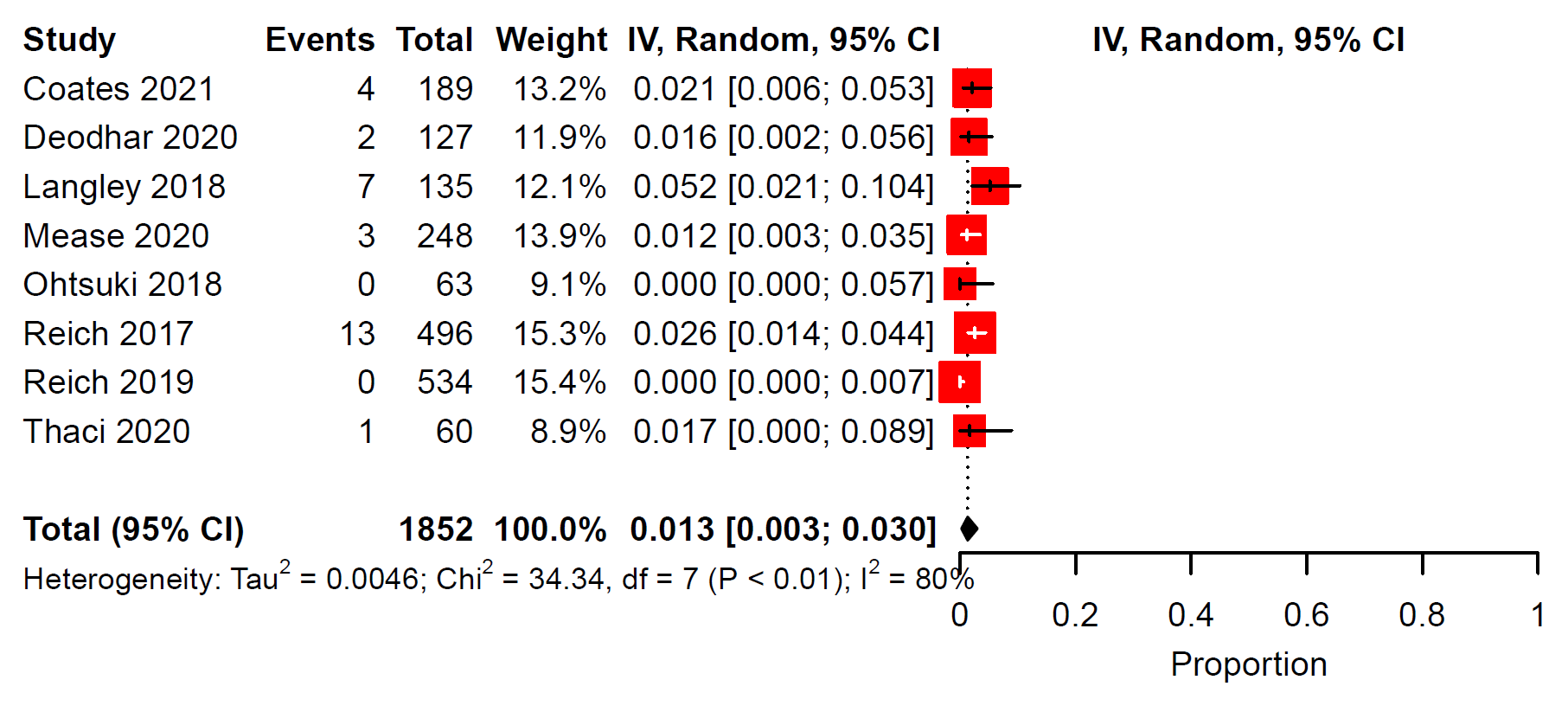


1. Omalizumab


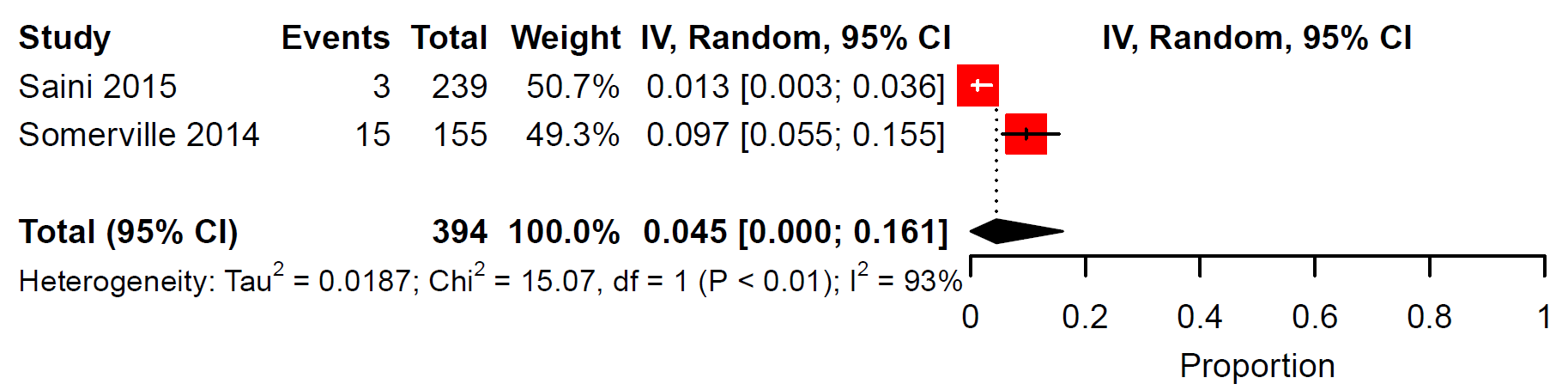


1. Risankizumab


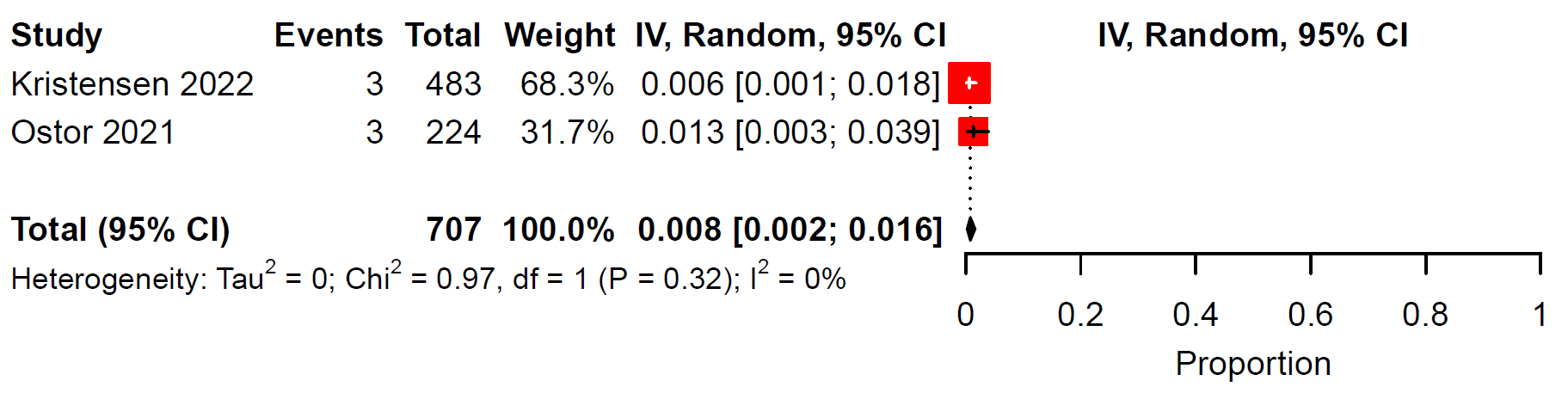


1. Sarilumab


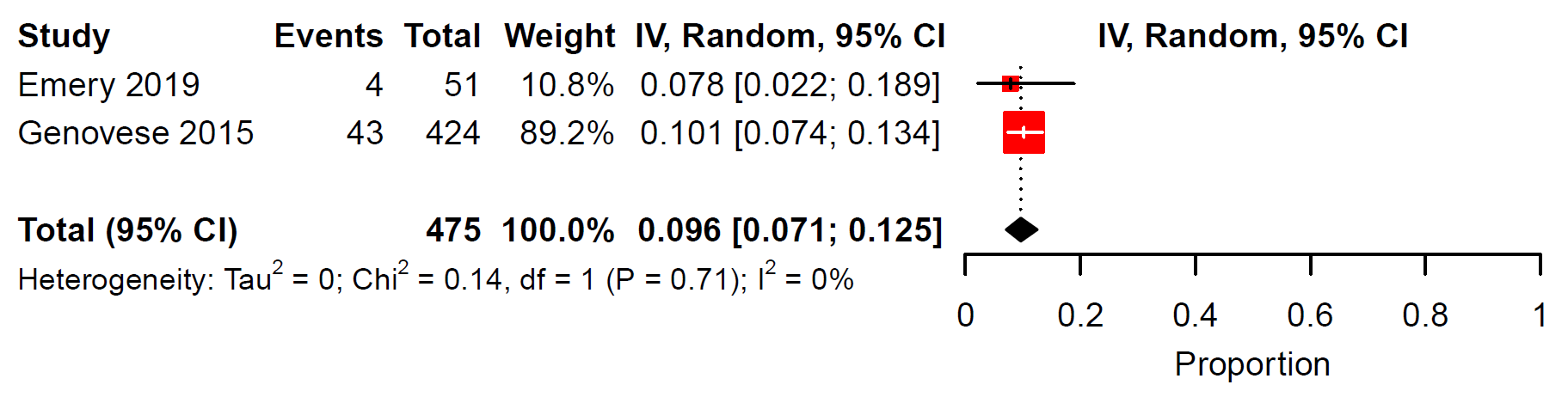


1. Secukinumab


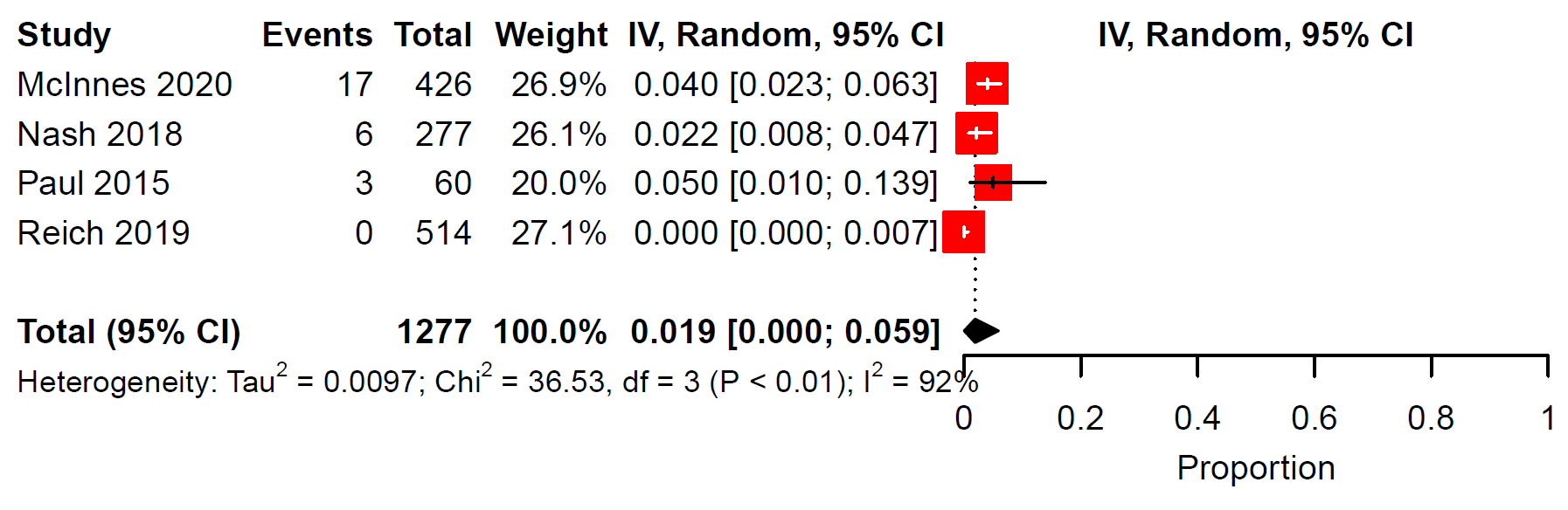


1. Tocilizumab


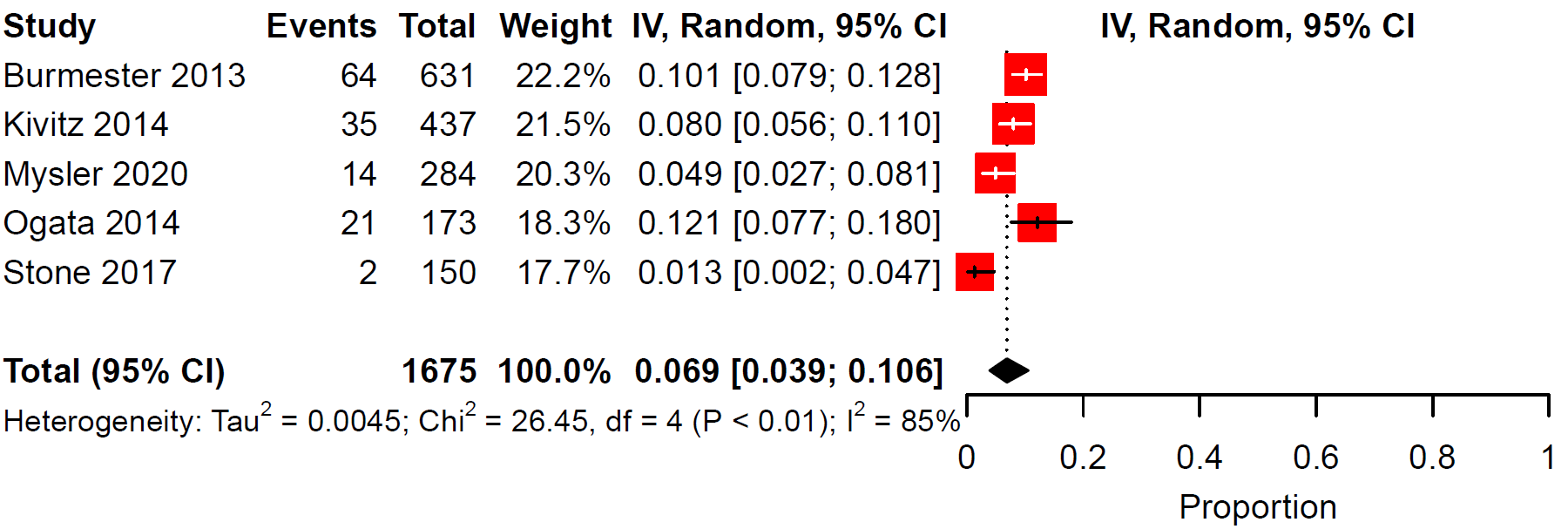


Supplementary File 4: Forest plots pooled prevalence of injection site reactions for a) Brodalumab, b) Canakinumab, c) Dupilumab, d) Golimumab, e) Guselkumab, f) Omalizumab, g) Risankizumab, h) Sarilumab, i) Secukinumab, and j) Tocilizumab.
